# Superatomic icosahedral-C_*n*_B_12-*n*_ (*n* = 0, 1, 2) Stuffed mononuclear and binuclear borafullerene and borospherene nanoclusters with spherical aromaticity

**DOI:** 10.1038/s41598-022-21809-w

**Published:** 2022-11-17

**Authors:** Min Zhang, Wei-Ping Jia, Ting Zhang, Bin-Bin Pei, Jia Xu, Xinxin Tian, Hai-Gang Lu, Si-Dian Li

**Affiliations:** Institute of Molecular Science, Shanxi University, Taiyuan, 030006 China

**Keywords:** Chemistry, Materials science, Nanoscience and technology

## Abstract

Boron and boron-based nanoclusters exhibit unique structural and bonding patterns in chemistry. Extensive density functional theory calculations performed in this work predict the mononuclear walnut-like *C*_*i*_ C_50_B_54_ (**1**) (C_2_B_10_@C_48_B_44_), *C*_1_ C_50_B_54_ (**2**) (CB_11_@C_49_B_43_), and *S*_10_ C_50_B_54_ (**3**) (B_12_@C_50_B_42_) which contain one icosahedral-C_*n*_B_12-*n*_ core (*n* = 0, 1, 2) at the center following the Wade’s skeletal electron counting rules and the approximately electron sufficient binuclear peanut-like *C*_*s*_ C_88_B_78_ (**4**) ((C_2_B_10_)_2_@C_84_B_58_), *C*_*s*_ C_88_B_78_ (**5**) ((CB_11_)_2_@C_86_B_56_), *C*_*s*_ C_88_B_78_ (**6**) ((B_12_)_2_@C_88_B_54_), *C*_*s*_ B_180_ (**7**) ((B_12_)_2_@B_156_), *C*_*s*_ B_182_ (**8**) ((B_12_)_2_@B_158_), and *C*_*s*_ B_184_ (**9**) ((B_12_)_2_@B_160_) which encapsulate two interconnected C_*n*_B_12-*n*_ icosahedrons inside. These novel core–shell borafullerene and borospherene nanoclusters appear to be the most stable species in thermodynamics in the corresponding cluster size ranges reported to date. Detailed bonding analyses indicate that the icosahedral B_12_^2−^, CB_11_^−^, and C_2_B_10_ cores in these core–shell structures possess the superatomic electronic configuration of 1S^2^1P^6^1D^10^1F^8^, rendering spherical aromaticity and extra stability to the systems. Such superatomic icosahedral-C_*n*_B_12-*n*_ stuffed borafullerenes and borospherenes with spherical aromaticity may serve as embryos to form bulk boron allotropes and their carbon-boron binary counterparts in bottom-up approaches.

## Introduction

Boron (1s^2^2s^2^2p^1^) exhibits unique structures and bonding patterns in chemistry to compensate for its prototypical electron-deficiency^1^. Dicoordinated boranes and tricoordinated borylenes are found to possess special reactivities on dinitrogen (N_2_) activations in both recent experimental and theoretical investigations^2–4^. At least sixteen distinct bulk boron allotropes have been experimentally known to be predominately constructed by interconnected icosahedral-B_12_ cages that in many cases are accompanied by interstitial boron atoms lying outside the icosahedrons, the most widely accepted structural model of boron-rich boron carbide B_4_C has CB_11_ icosahedrons with C-B-C intericosahedral chains, while the most frequently encountered boranes and carboranes contain icosahedral-C_*n*_B_12-*n*_ skeletons (*n* = 0, 1, 2)^1,5–8^. Derivatives of icosahedral borane B_12_H_12_^2−^ and carborane C_2_B_10_H_12_ bound to tumor-specific antigens have been the main focus of interests in the area of boron neutron capture therapy (BNCT)^1^ and globular B_12_Br_12_^2−^ was recently found to function as anionic inorganic membrane carriers for a broad range of hydrophilic cargo molecules^9^, further indicating the importance of *closo*-C_*n*_B_12-*n*_ icosahedrons in boron chemistry and materials science. In contrast, persistent joint photoelectron spectroscopy (PES) and first-principles theory investigations in the past two decades have shown that size-selected B_*n*_^−/0^ nanoclusters exhibit a great structural diversity in an unexpectedly wide size range, including the planar or quasi-planar (2D) boron clusters (*n* = 3–38, 41, 42) which provided experimental evidence for the viability of monolayer borophenes^10–12^, cage-like borospherenes *D*_2*d*_ B_40_^−/0^ and *C*_3_/*C*_2_ B_39_^13,14^ which were late extended to the B_*n*_^*q*^ borospherene family (*n* = 36–42, *q* = *n*−40) at first-principles theory level^15–18^, and bilayer *D*_2*h*_ B_48_^−/0^ which was recently expanded to the bilayer B_50_–B_72_ series and a bottom-up approach from medium-sized boron nanoclusters to bilayer borophenes at density functional theory (DFT)^19–24^. Seashell-like borospherenes *C*_2_ B_28_^−^ and *C*_*s*_ B_29_^−^ were observed in PES measurements as minor isomers of the systems^25,26^. Neutral fullerene-like *D*_2*d*_ B_14_ and double-ring tubular *D*_2*d*_ B_20_ have also been predicted at first-principles theory levels^27,28^. Inspired by the previously predicted icosahedral-B_12_ stuffed amorphous B_74_, B_84_, B_101_, and B_102_^29–32^ and based on the structural motif of *D*_5*h*_ C_70_ and extensive DFT calculations, our group recently reported the high-symmetry core–shell *C*_5*v*_ B_111_^+^ which satisfies the Wade’s *n* + 1 and *n* + 2 skeletal electron counting rules exactly and the approximately electron sufficient *C*_*s*_ B_111_, *C*_*s*_ B_112_, *C*_*s*_ B_113_, and *C*_*s*_ B_114_ which are the most stable neutral core–shell borospherenes with a superatomic icosahedral-B_12_ core at the center reported to date in the size range between B_68_-B_130_, with *C*_*s*_ B_112_ being the thermodynamically most favorite species in the series^33^. The newly proposed high-symmetry core–shell *T*_*h*_ B_96_^34^ appears to be about 0.020 eV atom^−1^ less stable than our *C*_*s*_ B_112_ and *C*_*s*_ B_113_ at DFT. However, core–shell borospherene nanoclusters with more than one B_12_ icosahedrons at the center still remain unknown in both experiments and theory, missing an important step to form bulk boron allotropes from medium-sized boron nanoclusters in bottom-up approaches.

Facile gas-phase formations of cage-like borafullerenes C_59_B and C_69_B by atomic exchange resulting from exposure of pristine C_60_ and C_70_ to boron vapor were firstly realized in 2013, with doubly and triply doped molecules, as well as C_56_B_4_ and higher doped fullerenes formed at lower abundances, depending on exposure time and the amount of B available for reaction^35^. Meanwhile, theoretical investigations on the structural and electronic properties of the borafullerenes C_60-*n*_B_*n*_ (*n* = 1–12) and core–shell borafullerene C_12_B_68_ have been reported in the literature^36,37^. Borafullerenes B_40_C_30_, B_40_C_40_, and B_40_C_50_ isovalent with C_60_, C_70_, and C_80_, respectively, have also been predicted at DFT^38^. Prasad and Jemmis considered the stability of core–shell borafullerenes (C_50_B_34_ and C_48_B_36_^2−^) based on Wade’s skeletal electron counting rules at DFT level^39^. Nevertheless, the thermodynamically most stable core–shell borafullerene nanoclusters stuffed with one or more than one C_*n*_B_12-*n*_ icosahedrons (*n* = 0, 1, 2) at the center have not been reported to date.

Keeping the inspiration in mind and based on extensive DFT calculations, we predict herein the walnut-like *C*_*i*_ C_50_B_54_ (**1**) (C_2_B_10_@C_48_B_44_), *C*_1_ C_50_B_54_ (**2**) (CB_11_@C_49_B_43_), *S*_10_ C_50_B_54_ (**3**) (B_12_@C_50_B_42_) based on the structural motif of *I*_*h*_ C_80_ which possess one C_*n*_B_12-*n*_ icosahedron (*n* = 0, 1, 2) at the center and the peanut-like *C*_*s*_ C_88_B_78_ (**4**), *C*_*s*_ C_88_B_78_ (**5**), *C*_*s*_ C_88_B_78_ (**6**), *C*_*s*_ B_180_ (**7**), *C*_*s*_ B_182_ (**8**), *C*_*s*_ B_184_ (**9**) which possess two interconnected icosahedral-C_*n*_B_12-*n*_ cores as the most stable species in the corresponding cluster size ranges reported to date. The icosahedral-C_*n*_B_12-*n*_ cores (*n* = 0, 1, 2) in these core–shell borafullerene and borospherene nanoclusters possess prototypical superatomic electronic configurations, rendering spherical aromaticity and extra stability to the systems.

### Computational procedures

Based on the structural motif of *I*_*h*_ C_80_, we manually constructed the initial structures of the icosahedral-C_*n*_B_12-*n*_ (*n* = 0, 1, 2) stuffed core–shell C_50_B_54_ clusters which follow the Wade’s *n* + 1 and *n* + 2 skeletal electron counting rules^1^ exactly (Fig. S1). However, locating the most stable isomer of such a medium-sized C–B binary cluster with huge numbers of possible positional isomers appeared to be a computationally daunting task. To solve the problem, we compiled the Fixed Motif Local Minimum Search (FMLMS) program in this work which includes random structural generations based on the designated structural motifs, symmetry recognitions using the Symmol code^40^, and structural similarity checks using the Ultrafast Shape Recognition (USR) approach^41,42^. Semi-empirical quantum mechanical calculations using the GFNn-xTB program were implemented to optimize the constructed structures from FMLMS initially and screen out the most concerned low-lying isomers, followed by structural optimizations using the CP2K software suite^43–45^. Such a procedure proved to work well in locating the recently reported most stable core–shell borospherenes of B_111_-B_114_ based on the structural motif of *D*_5*h*_ C_70_^33^. The low-lying isomers of the core–shell C_50_B_34_, C_50_B_44_, C_50_B_54_ and C_88_B_78_ binary nanoclusters based on the structural motifs of *I*_*h*_ C_60_, *D*_5*h*_ C_70_, *I*_*h*_ C_80_, and *D*_5*d*_ C_120_, were located using FMLMS in this work, respectively (Figs. S4, S5 and S7). Similar processes were implemented on the binuclear core–shell *C*_2_ B_172_, *C*_*s*_ B_176_, *C*_2_ B_178_, *C*_*s*_ B_180_, *C*_*s*_ B_182_, *C*_*s*_ B_184_, *C*_2_ B_186_, *C*_*s*_ B_188_, *C*_*s*_ B_190_, *C*_*s*_ B_192_ (Figs. 2b, S8) based on the structural pattern of *C*_2*v*_ C_110_ as an extension of the previously reported most stable mononuclear *C*_*s*_ B_112_^33^. The lowest-lying ten to twenty isomers were then fully optimized at both DFT-PBE0^46^ and TPSSh^47^ levels with the all-electron basis sets of 6-31G(d) for both C and B^48^ implemented in Gaussian 09 suite^49^, with the relative energies further refined for the first few competitive lowest-energy isomers at PBE0/6-311G(d)^46–48^. Extensive Born–Oppenheimer molecular dynamics (BOMD) simulations were implemented for 30 ps on *C*_*i*_ C_50_B_54_ (**1**) and *S*_10_ C_50_B_54_ (**3**) at 1500 K and *C*_*s*_ B_184_ (**9**) at 500 K using the CP2K program^45^ to verify their dynamic stability at high temperatures. Natural bonding orbital (NBO) analyses were performed using the NBO 6.0 program^50^. Nucleus-independent chemical shifts (NICS)^51,52^ were calculated at the centers of the C_*n*_B_12-*n*_ icosahedrons (*n* = 0, 1, 2) to assess the spherical aromaticity of core–shell systems. Detailed bonding analyses on *C*_*i*_ C_50_B_54_ (**1**), *S*_10_ C_50_B_54_ (**3**), *C*_*s*_ C_88_B_78_ (**4**), *C*_*s*_ B_182_ (**8**) and *C*_*s*_ B_184_ (**9**) were carried out using the adaptive natural density partitioning (AdNDP 2.0) method^53,54^ at the PBE0/6-31G level^46,48^. The Electron Density of Delocalized Bonds (EDDB) was calculated using the EDDB code^55,56^, with the EDDB isosurfaces generated using the visual molecular dynamics (VMD) software^57^ to visualize the distribution of delocalized bonds. The IR and Raman spectra of *C*_*i*_ C_50_B_54_ (**1**), *C*_*s*_ C_88_B_78_ (**4**), and *C*_*s*_ B_184_ (**9**) were theoretically simulated at PBE0/6-31G(d).

## Results

### Structures and stabilities

**Figure 1:**
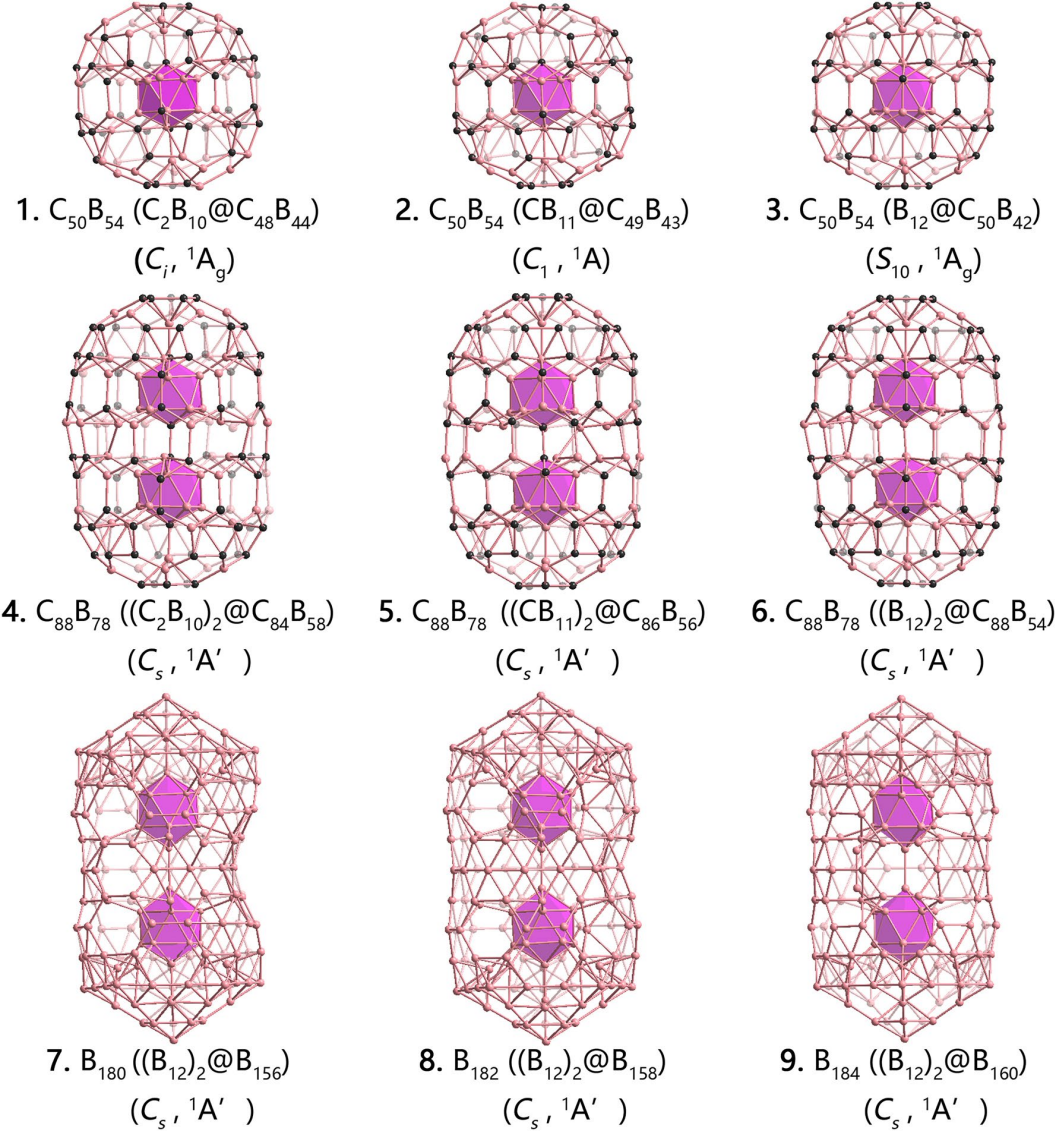
Optimized structures of *C*_*i*_ C_50_B_54_ (**1**), *C*_1_ C_50_B_54_ (**2**), *S*_10_ C_50_B_54_ (**3**), *C*_*s*_ C_88_B_78_ (**4**), *C*_*s*_ C_88_B_78_ (**5**), *C*_*s*_ C_88_B_78_ (**6**), *C*_*s*_ B_180_ (**7**), *C*_*s*_ B_182_ (**8**), and *C*_*s*_ B_184_ (**9**) at PBE0/6-311G(d) level, with the icosahedral-C_*n*_B_12-*n*_ (*n* = 0, 1, 2) cores at the centers highlighted in purple.

The structural constructions of mononuclear C_50_B_54_ (**1, 2, 3**), binuclear *C*_*s*_ C_88_B_78_ (**4, 5, 6**), and binuclear B_180_ (**7**), B_182_ (**8**)*,* and B_184_ (**9**) starting from the structural motifs of the corresponding fullerenes are illustrated in Figs. S1, S2 and S3, respectively. The optimized core–shell borafullerenes *C*_*i*_ C_50_B_54_ (**1**) (C_2_B_10_@C_48_B_44_), *C*_1_ C_50_B_54_ (**2**) (CB_11_@C_49_B_43_), and *S*_10_ C_50_B_54_ (**3**) (B_12_@C_50_B_42_) with one icosahedral-C_*n*_B_12-*n*_ (*n* = 0, 1, 2) core at the center, core–shell borafullerenes *C*_*s*_ C_88_B_78_ (**4**) ((C_2_B_10_)_2_@C_84_B_58_), *C*_*s*_ C_88_B_78_ (**5**) ((CB_11_)_2_@C_86_B_56_), *C*_*s*_ C_88_B_78_ (**6**) ((B_12_)_2_@C_88_B_54_) with two interconnected icosahedral-C_*n*_B_12-*n*_ (*n* = 0, 1, 2) cores, and core–shell borospherenes *C*_*s*_ B_180_ (**7**) ((B_12_)_2_@B_156_), *C*_*s*_ B_182_ (**8**) ((B_12_)_2_@B_158_), and *C*_*s*_ B_184_ (**9**) ((B_12_)_2_@B_160_) with two interconnected icosahedral-B_12_ cores are collectively shown in Fig. 1, with more alternative low-lying isomers obtained for C_50_B_54_, C_88_B_78_, and B_184_ depicted in Figs. S4, S5 and S6, respectively.

**Figure 2:**
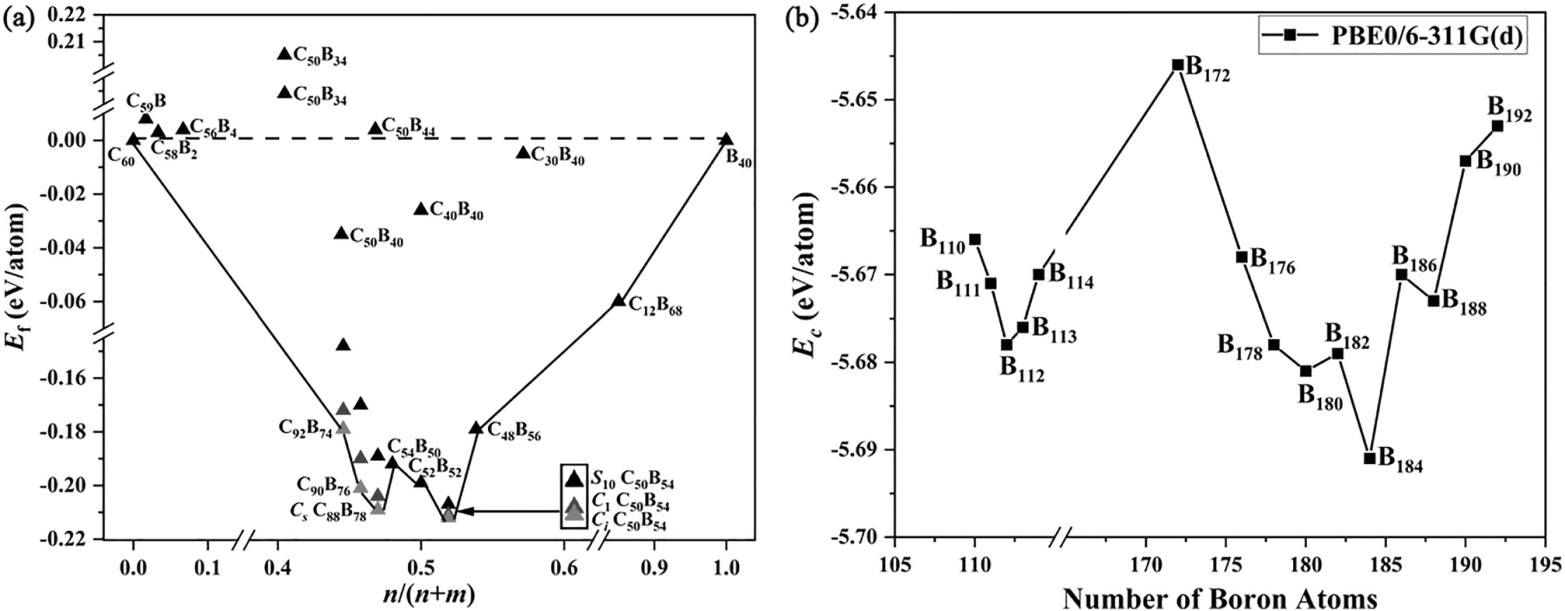
(**a**) Calculated formation energy per atom (*E*_f_, eV atom^−1^) as a function of the *n*/(*m* + *n*) ratio in the optimized boron–carbon clusters C_*m*_B_*n*_ and (**b**) cohesive energy per atom (*E*_c_, eV atom^−1^) of the optimized core–shell boron clusters B_*n*_ (*n* = 110–192) as a function of the cluster size (*n*) at PBE0/6-311G(d).

The calculated formation energies per atom *E*_f_ = (*E*_t_−*mμ*_B_−*nμ*_C_)/(*m*+ *n*) for the C_*m*_B_*n*_ borafullerenes are diagrammatically shown in Fig. 2a where *E*_t_, *μ*_B_ = *E*B_40_/40, and *μ*_C_ = *E*C_60_/60 are the total energy of C_*m*_B_*n*_ binary clusters and chemical potentials of the experimentally observed *D*_2*d*_ B_40_^13^ and *I*_*h*_ C_60_, respectively, while the cohesive energies per atom *E*_c_ = (*E*_t_−*nE*)/*n*) for B_*n*_ core–shell borospherenes are depicted in Fig. 2b where *E* is the energy of a free B atom in vacuum. The calculated nucleus-independent chemical shift (NICS) values at the geometric centers of the C_*n*_B_12-*n*_ (*n* = 0, 1, 2) icosahedral cores of the concerned core–shell borafullerenes and borospherenes and their HOMO–LUMO gaps (△*E*_gap_) at PBE0/6-311G(d) are comparatively tabulated in Tables S1 and S2.

As shown in Fig. S1, with one *closo*-B_12_ icosahedron located at the center and twelve *nido*-B_6_ pentagonal pyramids symmetrically distributed on the cage surface, the high-symmetry core–shell *I*_*h*_ B_104_ (B_12_@B_92_) based on the structural motif of *I*_*h*_ C_80_ is deficient by 50 electrons according to the Wade’s *n* + 1 and *n* + 2 skeleton electron counting rules and should therefore not be expected to be stable in thermodynamics^32,39,58^. *I*_*h*_ B_104_ can be made electron sufficient by substitution of 50 B atoms on the cage surface with 50 C atoms. Eighteen such low-lying walnut-like C_50_B_54_ positional isomers within 1.91 eV were obtained using FMLMS in Fig. S4. The high-symmetry *S*_10_ C_50_B_54_ (B_12_@C_50_B_42_) (**3**) (Fig. 1) as the ninth lowest-lying isomer possesses an almost ideal *closo*-B_12_ icosahedron at the center, ten *nido*-C_4_B_2_ pentagonal pyramids symmetrically distributed on the waist, and two *nido*-C_5_B pentagonal pyramids on the top and bottom. The core–shell *C*_1_ C_50_B_54_ (CB_11_@C_49_B_43_) (**2**) can be obtained by replacing the central B_12_ core in C_50_B_54_ (**3**) with a *closo*-CB_11_ icosahedron, with the top *nido*-C_5_B simultaneously changed into a *nido*-C_4_B_2_ pentagonal pyramid. The most stable C_50_B_54_ (C_2_B_10_@C_48_B_44_) (**1**) contains an icosahedral *closo*-C_2_B_10_ core at the center and twelve *nido*-C_4_B_2_ pentagonal pyramids evenly distributed on the cage surface in an overall symmetry of *C*_*i*_. C_50_B_54_ (**1**), C_50_B_54_ (**2**), and C_50_B_54_ (**3**) prove to be true minima on the potential surface of C_50_B_54_ with the smallest vibrational frequencies of *v*_min_ = 230.2, 222.4, and 208.4 cm^−1^ at PBE0/6-31G(d), respectively.

As shown in Fig. 2a and Table S1, as one of the two local minima on the formation energy *E*_f_ ~ *n*/(*n* + *m*) curve, *C*_*i*_ C_50_B_54_ (**1**) is the most stable core–shell borafullerene obtained to date, with the average formation energy per atom of *E*_f_ = − 0.213 eV atom^−1^ with respect to the experimentally known C_60_ and B_40_. It is 0.11 eV more stable than the second lowest-lying *C*_1_ C_50_B_54_ (**2**) and 0.58 eV more stable than the ninth lowest-lying *S*_10_ C_50_B_54_ (**3**) at PBE0/6-311G(d) level (Fig. S4). Other approximately electron sufficient close-lying species *C*_*i*_ C_48_B_56_ (B_12_@C_48_B_44_), *C*_2_ C_52_B_52_ (B_12_@C_52_B_40_), and *C*_2_ C_54_B_50_ (B_12_@C_54_B_38_) all appear to be obviously less favorable in thermodynamics than *C*_*i*_ C_50_B_54_ (**1**). The seventeenth high-symmetry isomer *C*_5_ C_50_B_54_ in the structural motif of *D*_5*h*_ C_80_ with a B_12_ icosahedron at the center lies 1.87 eV less stable than C_50_B_54_ (**1**) (Fig. S1). As indicated in Table S1, C_50_B_54_ (**1/2/3**) have the largest calculated HOMO–LUMO gaps of ∆*E*_gap_ = 2.24/2.28/2.75 eV in the low-lying core–shell borafullerene series obtained in this work, well supporting the high chemical stabilities of these mononuclear core–shell borafullerenes. The previously predicted electron sufficient core–shell *C*_2*h*_ C_50_B_34_^39^ in the structural motif of *I*_*h*_ C_60_ (which was distorted to a more stable *C*_1_ C_50_B_34_ obtained in this work, Fig. S7), core–shell *C*_5_ C_50_B_44_ in the structural motif of *D*_5*h*_ C_70_ obtained in this work (Fig. S7), and the previously reported cage-like borafullerenes C_30_B_40_, C_40_B_40_, and C_50_B_40_^38^ all appear to be obviously less favorable than C_50_B_54_ (**1**) (Fig. 2a). It is also noticed that C_50_B_54_ (**1**), C_50_B_54_ (**2**), and C_50_B_54_ (**3**) are all considerably more favorable in formation energies than the experimentally observed C_59_B, C_58_B_2_, and C_56_B_4_ and theoretically predicted amorphous core–shell *C*_1_ C_12_B_68_^35–37^.

The walnut-like core–shell borafullerenes C_50_B_54_ (**1**, **2**, **3**) can be extended in axial dimension to form the approximately electron sufficient peanut-like *C*_*s*_ C_88_B_78_ (**4**) ((C_2_B_10_)_2_@C_84_B_58_), *C*_*s*_ C_88_B_78_ (**5**) ((CB_11_)_2_@C_86_B_56_), *C*_*s*_ C_88_B_78_ (**6**) ((B_12_)_2_@C_88_B_54_) based on the structural framework of *D*_5*d*_ C_120_ which contain two interconnected icosahedral C_2_B_10_, CB_11_, and B_12_ cores inside the outer shells, respectively (Figs. 1 and S2). *C*_*s*_ C_88_B_78_ (**4**) as the second local minimum on the *E*_f_ ~ *n*/(*n* + *m*) curve (Fig. 2a) with *E*_f_ = − 0.209 eV atom^−1^ appears to be 0.005 and 0.020 eV atom^−1^ more stable than C_88_B_78_ (**5**) and *C*_*s*_ C_88_B_78_ (**6**) in formation energy, respectively, indicating again that icosahedral-C_2_B_10_ cores are better favored in energy over both CB_11_ and B_12_ icosahedrons in core–shell borafullerenes. The electron-precise C_92_B_74_ and approximately electron sufficient C_90_B_76_ with two interconnected icosahedral-C_*n*_B_12-*n*_ cores (*n* = 0, 1, 2) appear to be slightly less stable in thermodynamics than their C_88_B_78_ (**4**) counterpart (Fig. 2a). The prediction of mononuclear C_50_B_54_ (**1**, **2**, **3**) and binuclear C_88_B_78_ (**4**, **5**, **6**) as the two minima on the *E*_f_ ~ *n*/(*n* + *m*) curve indicates that *I*_*h*_ C_80_ and its expanded fullerene analog *D*_5*d*_ C_120_ provide the right cavities and optimum structural motifs to form core–shell borafullerenes with one and two icosahedral-C_*n*_B_12-*n*_ (*n* = 0, 1, 2) cores (Fig. 2a), respectively. In contrast, the structural motifs generated from both *I*_*h*_ C_60_ and *D*_5*h*_ C_70_ appear to be too small in size to host icosahedral-C_*n*_B_12-*n*_ (*n* = 0, 1, 2) cores comfortably in core–shell borafullerenes, as demonstrated in the cases of core–shell *C*_2*h*_/*C*_1_ C_50_B_34_ and *C*_5_ C_50_B_44_ (Figs. 2a and S7).

As an extension of the previously reported most stable mononuclear *C*_*s*_ B_112_ based on the framework of *D*_5*h*_ C_70_^33^, a series of binuclear core–shell borospherenes B_172_-B_192_ with two interconnected B_12_ icosahedrons at the center based on the structural motif of *C*_2*v*_ C_110_ are obtained in this work (Figs. 2b and S3). The almost electron-sufficient *C*_*s*_ B_188_ with the cohesive energy of *E*_c_ = − 5.673 eV atom^−1^ (Table S2) appears to be a local minimum on the *E*_c_ ~ *n* curve, but it is obviously less stable in thermodynamics than the approximately electron-sufficient *C*_*s*_ B_180_ (**7**) ((B_12_)_2_@B_156_), *C*_*s*_ B_182_ (**8**) ((B_12_)_2_@B_158_), and *C*_*s*_ B_184_ (**9**) ((B_12_)_2_@B_160_) which all lie within a deeper local minimum with *E*_c_ = − 5.681, − 5.679, and − 5.691 eV atom^−1^ at PBE0, respectively (Fig. 2b and Table S2). *C*_*s*_ B_184_ (**9**) as the most stable species on the *E*_c_ ~ *n* curve contains two *closo*-B_12_ icosahedral cores doubly bound to an interstitial B_2_ unit. It is even more stable than the previously reported mononuclear *C*_*s*_ B_112_ where *E*_c_ = − 5.678 eV atom^−1^ at the same theoretical level^33^. Similar results are obtained at TPSSh/6-311G(d) in Fig. S8 where *C*_*s*_ B_184_ also appears to be the most stable species in cohesive energy in the size range between B_110_ and  B_192_. Binuclear B_184_ (**9**) with two icosahedral-B_12_ cores and one interstitial B_2_ unit is therefore the most stable core–shell borospherene reported to date in thermodynamics.

Extensive BOMD simulations provide strong evidence to support the dynamic stability of these core–shell nanoclusters. As demonstrations in Fig. S9, the thermodynamically stable *C*_*i*_ C_50_B_54_ (**1**), *S*_10_ C_50_B_54_ (**3**), and *C*_*s*_ B_184_ (**9**) were highly dynamically stable at 1500 K, 1500 K, and 500 K, with the small calculated average root-mean-square-deviations of RMSD = 0.10, 0.10, 0.07 Å and maximum bond length deviations of MAXD = 0.37, 0.34, and 0.31 Å, respectively. No other low-lying isomers were observed during the dynamical simulations in 30 ps.

### Bonding pattern analyses

**Figure 3:**
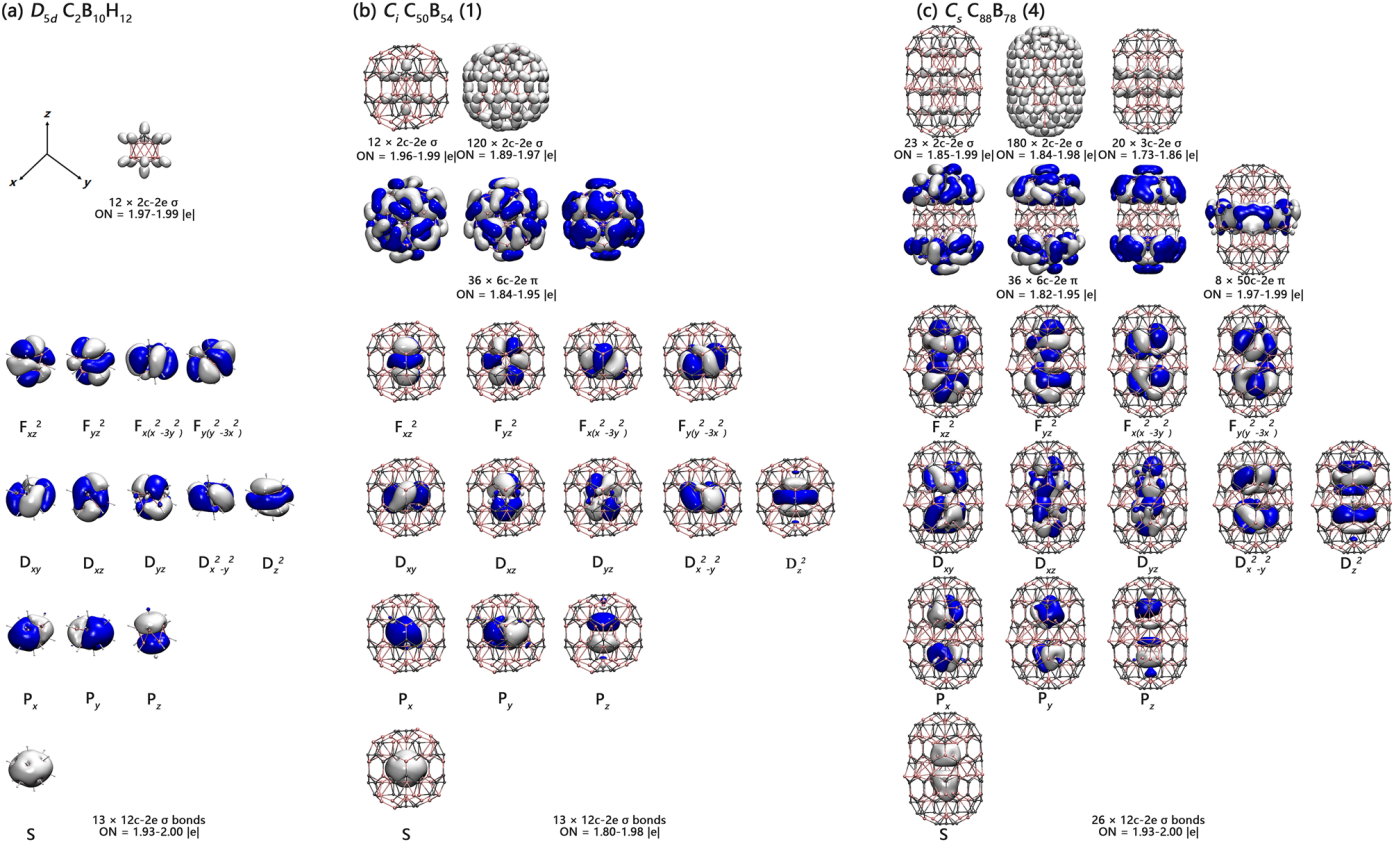
AdNDP bonding patterns of (**a**) *D*_5*d*_ C_2_B_10_H_12_, (**b**) *C*_*i*_ C_50_B_54_ (**1**), and (**c**) *C*_*s*_ C_88_B_78_ (**4**) with the occupation numbers (ONs) indicated.

The high stability of these core–shell nanoclusters originates from their unique electronic structures and bonding patterns. As demonstrations, detailed AdNDP bonding analyses on both closed-shell *C*_*i*_ C_50_B_54_ (**1**) and *C*_*s*_ C_88_B_78_ (**4**) are presented in Fig. 3. The icosahedral-C_2_B_10_ cores in both C_50_B_54_ (**1**) and C_88_B_78_ (**4**) are connected to the outer shells through radial B–B and C–B bonding interactions. To better understand the bonding nature of these binary core–shell structures, detailed bonding analysis on the prototypical carborane *D*_5*d*_ C_2_B_10_H_12_ is performed in Fig. 3a first. As expected, C_2_B_10_H_12_ possesses 12 2c-2e σ bonds in radial directions perpendicular to the cage surface, including 10 2c-2e B–H σ bonds on the waist and 2 2c-2e C–H σ bonds on the top and bottom with the occupation numbers of ON = 1.97–1.99 |e|. The remaining 26 valence electrons are distributed in 13 12c-2e delocalized bonds over the whole *D*_5*d*_ icosahedral*-*CB_10_C skeleton with ON = 1.93–2.00 |e|, including 1 12c-2e S-type bond, 3 12c-2e P-type bonds, 5 12c-2e D-type bonds, and 4 12c-2e F-type bonds. Such a bonding pattern well corresponds to the superatomic electronic configuration 1S^2^1P^6^1D^10^1F^8^ of *D*_5*d*_ C_2_B_10_H_12_ (Fig. S10) which is spherically aromatic in nature, as evidenced by the negative calculated NICS = − 29.22 ppm at the cage center.

The bonding pattern of *C*_*i*_ C_50_B_54_ (**1**) in Fig. 3b well demonstrates the superatomic behavior of its *C*_*i*_ icosahedral-CB_10_C core. C_50_B_54_ (**1**) contains 10 2c-2e B–B bonds and 2 2c-2e C–B σ bonds in radial directions between the CB_10_C icosahedron and outer shell to saturate the dangling valences of icosahedral core, 120 B-B or B-C or C–C 2c-2e σ bonds on the cage surface, and 36 6c-2e π bonds on 12 *nido*-C_4_B_2_ pentagonal pyramids in the first row, with 3 6c-2e π bonds over each C_4_B_2_ pentagonal pyramid matching the 4*n* + 2 aromatic rule with *n* = 1 (suggesting the existence of local π-aromaticity over each C_4_B_2_ pentagon in on the surface of C_50_B_54_ (**1**), similar to the situation in benzene C_6_H_6_). Its remaining 13 12c-2e bonds are delocalized over the whole *closo*-CB_10_C icosahedral core, including 1 12c-2e S-type bond, 3 12c-2e P-type bonds, 5 12c-2e D-type bonds, and 4 12c-2e F-type bonds, well corresponding to the 13 12c-2e delocalized bonds of *D*_*5d*_ C_2_B_10_H_12_ in Fig. 3a. Such a bonding pattern clearly indicates that the icosahedral-CB_10_C core in *C*_*i*_ C_50_B_54_ (**1**) possesses a typical superatomic electron configuration, similar to the situation in *D*_5*d*_ C_2_B_10_H_12_. Similar bonding patterns exist in *C*_1_ C_50_B_54_ (**2**) and *S*_10_ C_50_B_54_ (**3**) which contain negatively charged icosahedral-CB_11_^-^ and icosahedral-B_12_^2−^ cores, respectively (Fig. S11). The binuclear *C*_*s*_ C_88_B_78_ (**4**) possesses a similar but more complicated bonding pattern. As shown in Fig. 3c, C_88_B_78_ (**4**) contains 1 C–C 2c-2e σ bond between the two icosahedral-CB_10_C cores and 22 B-B or 2 C-B σ bonds in radial directions, 180 B-B or C-B or C–C 2c-2e σ bonds on the cage surface, and 20 3c-2e σ bonds on the waist between ten capping B atoms and the corresponding hexagonal holes on the surface in an overall symmetry of *C*_*s*_. In addition to the 36 6c-2e π bonds over 12 C_5_B or C_4_B_2_ pentagonal pyramids on the top and bottom, C_88_B_78_ (**4**) also possesses 8 50c-2e π bonds delocalized over the “girdle” composed of ten hexagonal pyramids on the waist in between. Most interestingly, with 26 12c-2e bonds over the CB_10_C-CB_10_C binuclear core in C_88_B_78_ (**4**), there exist 13 12c-2e bonds over each *closo*-CB_10_C icosahedron, including 1 12c-2e S-type bond, 3 12c-2e P-type bonds, 5 12c-2e D-type bonds, 4 12c-2e F-type bonds, well corresponding to the 13 12c-2e delocalized bonds of *D*_*5d*_ C_2_B_10_H_12_ in Fig. 3a. Thus, each *closo*-CB_10_C icosahedron in C_88_B_78_ (**4**) follows the superatomic electronic configuration of 1S^2^1P^6^1D^10^1F^8^, corresponding again to the 13 12c-2e delocalized bonds of *D*_*5d*_ C_2_B_10_H_12_ in Fig. 3a.

The local π-aromaticities over the twelve C_4_B_2_ pentagons and spherical aromaticities over each C_2_B_10_ icosahedral core in both C_50_B_54_ (**1**) and C_88_B_78_ (**4**) are also demonstrated in their calculated EDDB isosurface maps depicted in Fig. S13. The average values of atomic contribution of EDDB = 1.31, 1.33 *e* in the C_2_B_10_ icosahedrons in C_50_B_54_ (**1**) and C_88_B_78_ (**4**) are obviously larger than the corresponding values of EDDB = 0.93 and 1.00 *e* in the remaining parts, respectively, well supporting the spherical aromaticity of superatomic cores, while the observed high EDDB values over each C_4_B_2_ pentagon on the cage surface in continuous distributions indicate the existence of local π-aromaticity in the systems.

Such bonding patterns render spherical aromaticity to both *C*_*i*_ C_50_B_54_ (**1**) and *C*_*s*_ C_88_B_78_ (**4**), as evidenced by the negative calculated NICS = − 23.23 ppm and NICS = − 32.47, − 28.04 ppm at the cage centers of their C_2_B_10_ icosahedral cores, respectively. With the calculated NICS = − 17.70 ppm and NICS = − 32.68, − 32.65 ppm at the cage centers of their C_2_B_10_ and B_12_^2−^ icosahedral cores, respectively, both C_50_B_54_ (**3**) and B_184_ (**9**) also appear to be spherically aromatic in nature. Similar NICS values exist in the spherically aromatic C_50_B_54_ (**2**), C_50_B_54_ (**3**), C_88_B_78_ (**5**), *C*_*s*_ C_88_B_78_ (**6**), B_180_ (**7**), and B_182_ (**8**).

### IR and Raman spectral simulations

**Figure 4:**
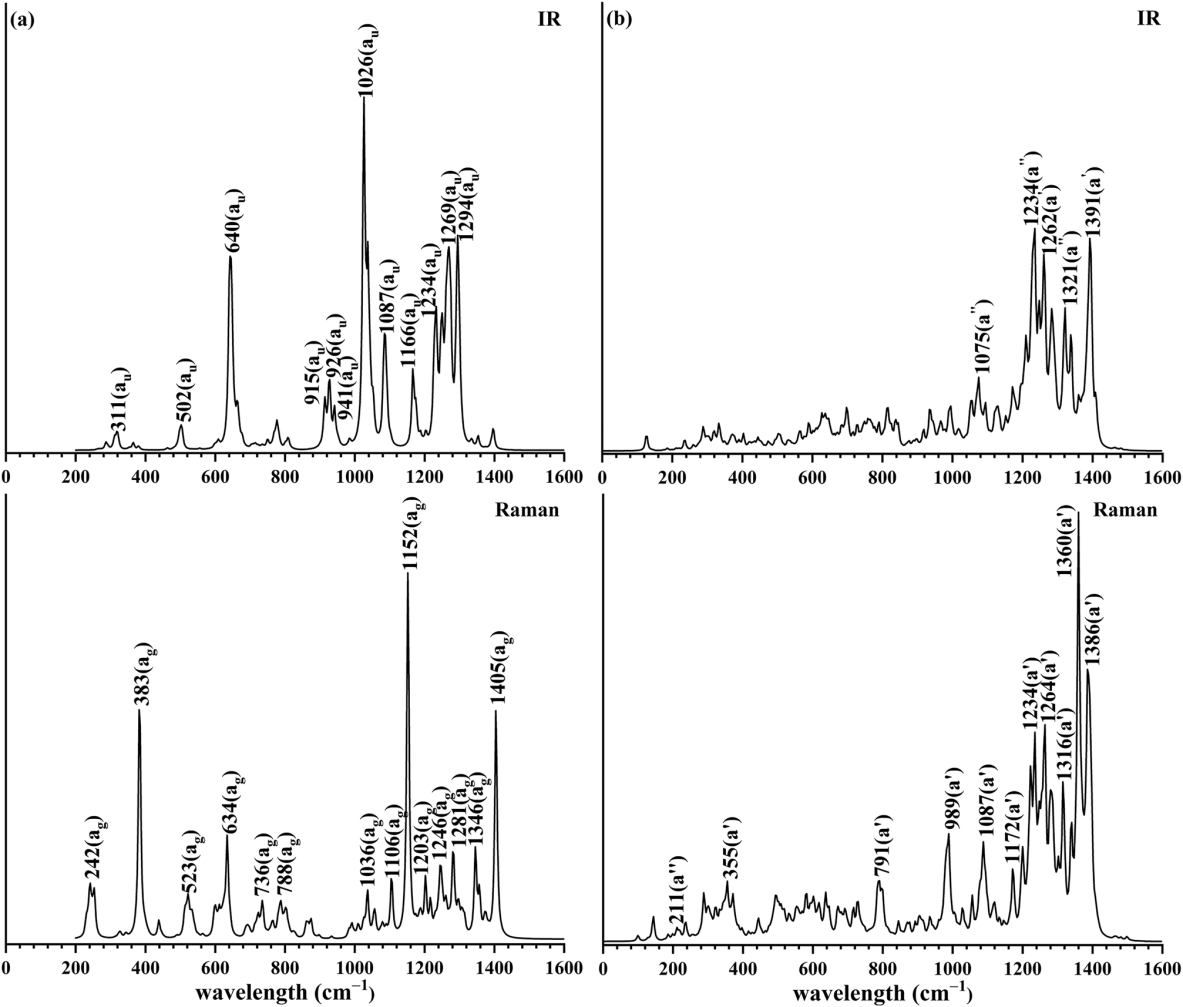
Simulated IR and Raman spectra of (**a**) *C*_*i*_ C_50_B_54_ (**1**) and (**b**) *C*_*s*_ C_88_B_78_ **(4**) at PBE0/6-31G(d) level.

The infrared (IR) and Raman spectra of *C*_*i*_ C_50_B_54_ (**1**) and *C*_*s*_ C_88_B_78_ (**4**) are computationally simulated at PBE0/6-31G(d) in Fig. 4 to facilitate their spectral characterizations. *C*_*i*_ C_50_B_54_ (**1**) exhibits three major IR peaks at 640 (a_u_), 1026 (a_u_), and 1294 cm^−1^ (a_u_), while *C*_*s*_ C_88_B_78_ (**4**) possesses two major IR peaks at 1234 (a’’) and 1391 cm^−1^ (a’), respectively. The three major Raman active peaks of C_50_B_54_ (**1**) occur at 383 (*a*_g_), 1152 cm^−1^ (*a*_g_), and 1405 cm^−1^ (*a*_g_), with the weak peak at 242 cm^−1^ (*a*_g_), strong peaks at 383 (*a*_g_), and strong peak at 1405 cm^−1^ (*a*_g_) representing typical “radial breathing modes” (RBMs) of the outer shell, the core + shell system as a whole, and the inner icosahedral-C_2_B_10_ core of *C*_*i *_C_50_B_54_ (**1**), respectively. Such RBMs can be used to characterize the hollow boron-based nanostructures in experiments^59^. Similarly, *C*_*s*_ C_88_B_78_ (**4**) exhibits two major Raman peaks at 1264 cm^−1^ (a’) and 1360 cm^−1^ (a’) and three RBM vibrational modes at 211 cm^−1^ (a’), 329 cm^-1^ (a’), 858 cm^-1^ (a’), respectively.

## Conclusions

The analyses above indicate that, based on the structural motifs of the related fullerenes and extensive DFT calculations, the mononuclear C_50_B_54_ (**1**, **2**, **3**) and binuclear *C*_*s*_ C_88_B_78_ (**4**, **5**, **6**), B_180_ (**7**), B_182_ (**8**), and B_184_ (**9**) nanoclusters obtained in this work with one or two icosahedral-C_*n*_B_12-*n*_ cores at the center are the most stable core–shell borafullerenes and borosphenrenes in thermodynamics in the corresponding cluster size ranges reported to date. The B_12_^2−^, CB_11_^-^, and C_2_B_10_ icosahedrons encapsulated in these core–shell nanostructures possess the superatomic electronic configurations (1S^2^1P^6^1D^10^1F^8^) of the experimentally known icosahedral *I*_*h*_ B_12_H_12_^2−^, *C*_*5*_ CB_11_H_12_^-^_,_ and *D*_*5d*_ C_*2*_B_10_H_12_, respectively, rendering prototypical spherical aromaticity to the systems. Theoretical investigations on core–shell borafullerenes and borospherenes with more than two superatomic icosahedral-C_*n*_B_12-*n*_ cores accompanied by suitable numbers of interstitial boron atoms are currently in progresses. Experimental investigations are invited to synthesize icosahedral-C_*n*_B_12-*n*_ stuffed core–shell borafullerenes and borospherenes to form bulk boron allotropes and their carbon-boron binary counterparts with novel electronic and mechanic properties in bottom-up approaches.

## Supplementary Information

Supplementary Information.

## Data Availability

The datasets used and/or analysed during the current study are available from the corresponding author on reasonable request.
