# Supplementary material for: Superatomic icosahedral-CnB12-n (n = 0, 1, 2) Stuffed mononuclear and binuclear borafullerene and borospherene nanoclusters with spherical aromaticity
Source: Sci Rep. 2022 Nov 17;12:19741. doi: 10.1038/s41598-022-21809-w (PMC9672116; doi:10.1038/s41598-022-21809-w)
Supplement: Supplementary file 1 — Supplementary Information. [file 41598_2022_21809_MOESM1_ESM.pdf]

Supporting Information

Superatomic Icosahedral-C_nB_{12-n} (n = 0, 1, 2) Stuffed Mononuclear and Binuclear Borafullerene and Borospherene Nanoclusters with Spherical Aromaticity

Min Zhang, Wei-Ping Jia, Ting Zhang, Bin-Bin Pei, Jia Xu, Xinxin Tian, Hai-Gang Lu*, and Si-Dian Li*

Institute of Molecular Science, Shanxi University, Taiyuan 030006, China

Contents

Figure S1 Structural constructions of mononuclear I_h B_{104}, C_i $C_{50}B_{54}$ (**1**), C_1 $C_{50}B_{54}$ (**2**), and S_{10} $C_{50}B_{54}$ (**3**) based on the structural motif of I_h C_{80}.

Figure S2 Structural constructions of binuclear C_s $C_{88}B_{78}$ (**4**), C_s $C_{88}B_{78}$ (**5**), and C_s $C_{88}B_{78}$ (**6**) based on the structural motifs of I_h C_{80} and D_{5d} C_{120}.

Figure S3 Structural constructions of binuclear C_s B_{180} (**7**), C_s B_{182} (**8**), and C_s B_{184} (**9**) based on the structural motifs of I_h C_{70} and C_{2v} C_{110}.

Figure S4 Relative energies of low-lying isomers of $C_{50}B_{54}$.

Figure S5 Relative energies of low-lying isomers of $C_{88}B_{78}$.

Figure S6 Relative energies of low-lying isomers of B_{184}.

Figure S7 Optimized structures of C_1 $C_{50}B_{34}$ and C_{2h} $C_{50}B_{34}$ based on the structural motif of I_h C_{60} and optimized structure of C_5 $C_{50}B_{44}$ based on the structural motif of D_{5h} C_{70} .

Figure S8 Calculated cohesive energy per atom (E_c, eV/atom) of the optimized core-shell boron clusters B_n (n = 110-192) as a function of the cluster size (n) at TPSSh/6-311G(d) level.

Figure S9 BOMD simulations of (a) C_i $C_{50}B_{54}$ (**1**) at 1500K, (b) S_{10} $C_{50}B_{54}$ (**3**) at 1500K, and (c) C_s B_{184} (**9**) at 500K.

Figure S10 The superatomic electronic configuration $1S^2 1P^6 1D^{10} 1F^8$ of carborane D_{5d} $C_2B_{10}H_{12}$.

Figure S11 AdNDP bonding analysis of S_{10} $C_{50}B_{54}$ (**3**).

Figure S12 Superatomic bonding orbitals of the two icosahedral B_{12} cores in C_s B_{182} (8) and C_s B_{184} (**9**).

Figure S13 Isosurfaces of the electron densities of delocalized bonds (EDDB) of C_i $C_{50}B_{54}$ (**1**) (a) and C_s $C_{78}B_{88}$ (**4**) (b), with the isovalues and average values of atomic contribution indicated.

Table S1 Calculated formation energies per atom and HOMO-LUMO gaps of the optimized borafullerenes $C_{50}B_{54}$ (**1**, **2**, **3**) and $C_{88}B_{78}$ (**4**, **5**, **6**) and the calculated NICS values (in ppm) at the geometric centers of the

icosahedral-C_nB_{12-n} cores in them.

Table S2 Calculated cohesive energies per atom and HOMO-LUMO gaps of the optimized core-shell borospherenes B_n (n = 176-188) and the calculated NICS values (in ppm) at the geometric centers of icosahedral-B_{12} cores in them.

Table S3 Optimized coordinates (x, y, z) of C_i $C_{50}B_{54}$ (**1**), C_1 $C_{50}B_{54}$ (**2**), S_{10} $C_{50}B_{54}$ (**3**), C_s $C_{88}B_{78}$ (**4**), C_s $C_{88}B_{78}$ (**5**), C_s $C_{88}B_{78}$ (**6**), C_s B_{180} (**7**), C_s B_{182} (**8**) and C_s B_{184} (**9**) at PBE0/6-311G(d) level.

Fig. S1 Structural constructions of mononuclear I_h B_{104}, C_i $C_{50}B_{54}$ (**1**)，C_1 $C_{50}B_{54}$ (**2**)，and S_{10} $C_{50}B_{54}$ (**3**) based on the structural motif of I_h C_{80}, with the icosahedral-C_nB_{12-n} (n = 0, 1, 2) cores at the center highlighted in pink.

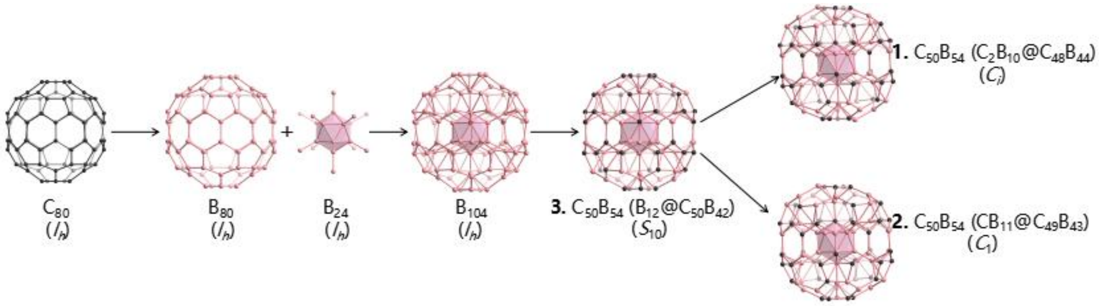

Fig. S2 Structural constructions of binuclear C_s $C_{88}B_{78}$ (**4**)，C_s $C_{88}B_{78}$ (**5**)，and C_s $C_{88}B_{78}$ (**6**) based on the structural motifs of I_h C_{80} and D_{5d} C_{120}, with the interconnected icosahedral-C_nB_{12-n} (n = 0, 1, 2) cores at the center highlighted in pink.

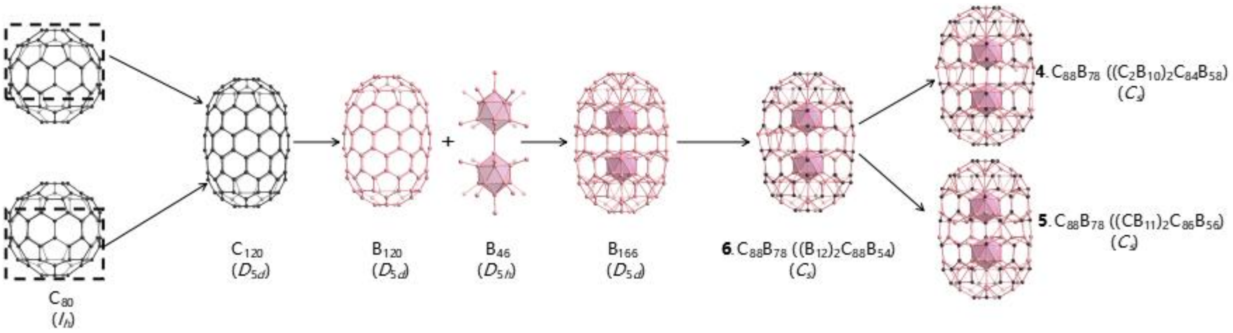

Fig. S3 Structural constructions of binuclear C_s B_{180} (**7**), C_s B_{182} (**8**), and C_s B_{184} (**9**) based on the structural motifs of I_h C_{70} and C_{2v} C_{110}, with the interconnected icosahedral-B_{12} (n = 0, 1, 2) cores at the center highlighted in pink.

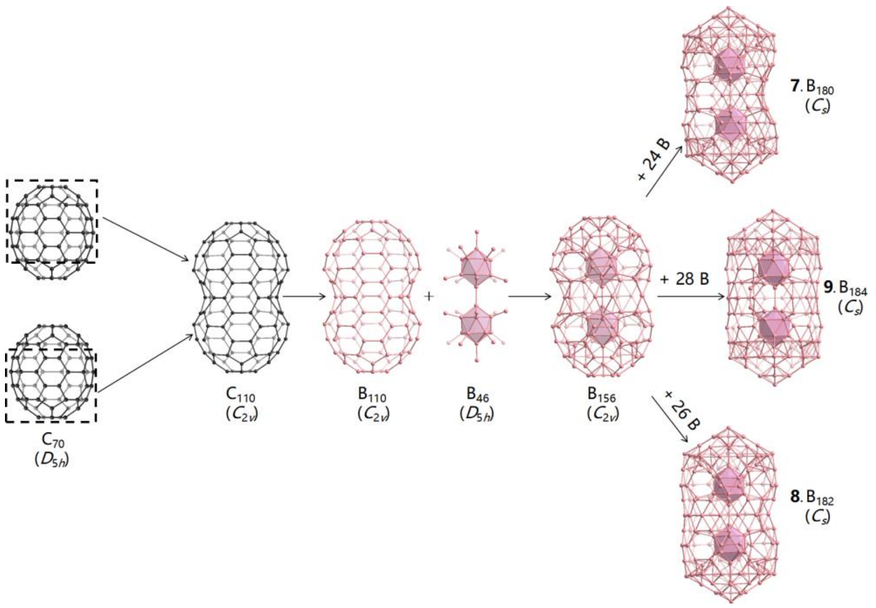

Fig. S4 Relative energies of the low-lying isomers of $C_{50}B_{54}$ at PBE0/6-31G(d) and PBE0/6-311G(d) (in parentheses) levels in eV.

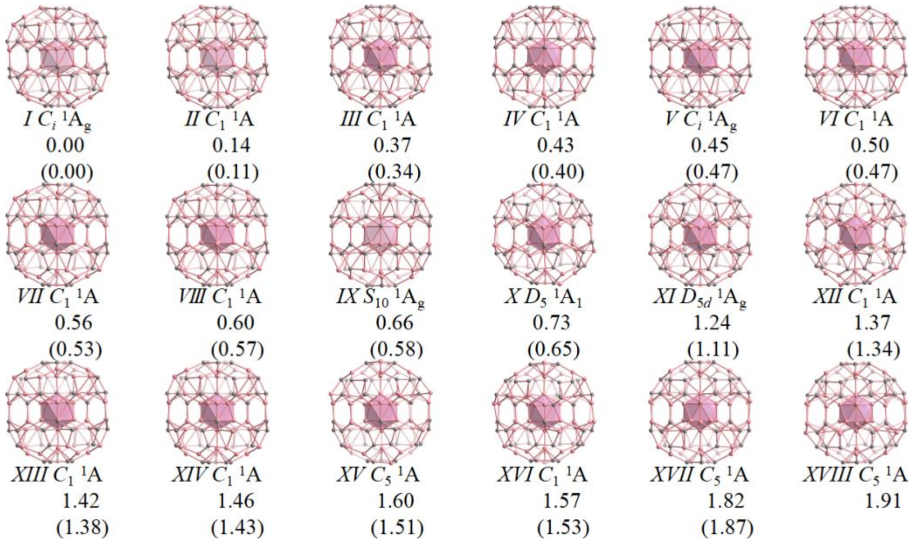

Fig. S5 Relative energies of the low-lying isomers of $C_{88}B_{78}$ at PBE0/6-31G(d) and PBE0/6-311G(d) (in parentheses) levels in eV.

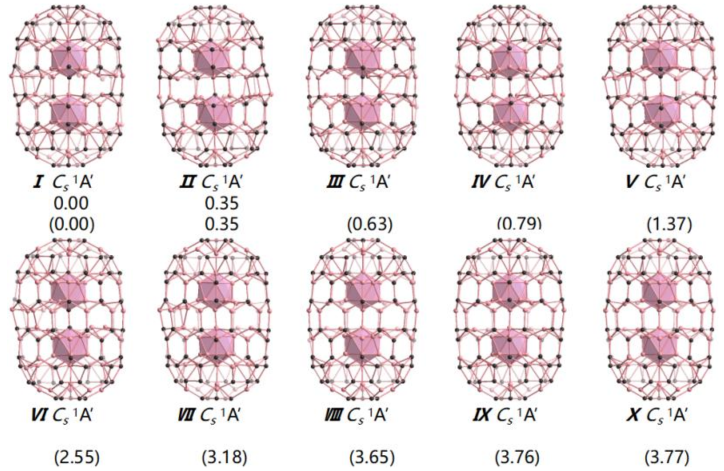

Fig. S6 Relative energies of the low-lying isomers of B_{184} at PBE0/6-31G(d) and PBE0/6-311G(d) (in parentheses) levels in eV.

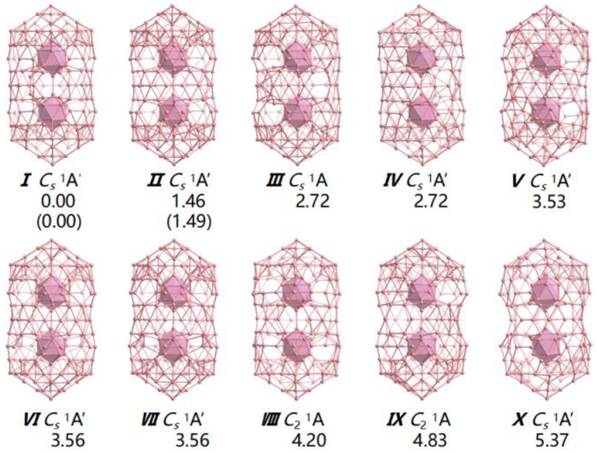

Fig. S7 (a) Optimized structures of C_1 $C_{50}B_{34}$ obtained in this work and C_{2h} $C_{50}B_{34}$ from Ref. 41 based on the structural motif of I_h C_{60}, with relative energies indicated in eV. (b) Optimized structure of C_5 $C_{50}B_{44}$ constructed in the work based on the structural motif of D_{5h} C_{70} at PBE0/6-311G(d) level.

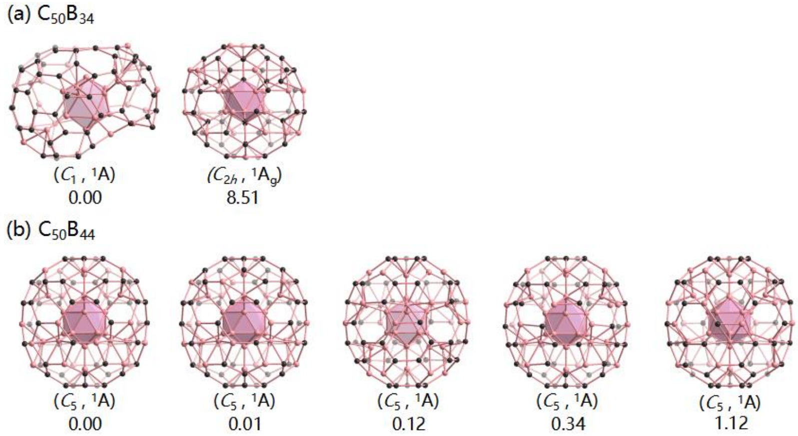

Fig. S8 Calculated cohesive energy per atom (E_c, eV/atom) of the optimized core-shell boron clusters B_n (n = 110-192) as a function of the cluster size (n) at TPSSh/6-311G(d) level.

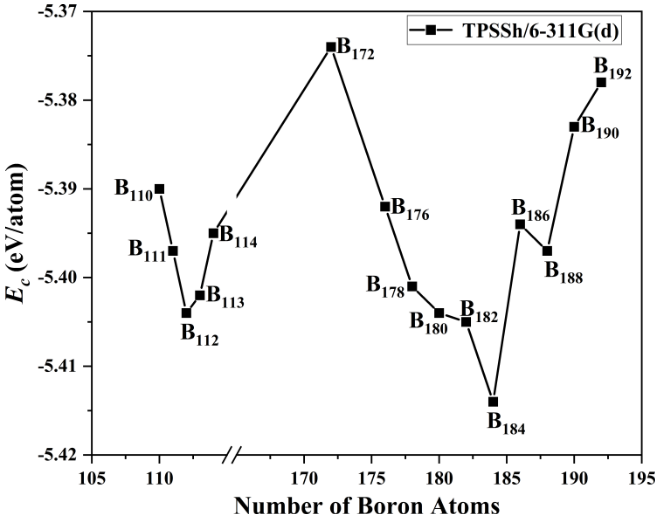

Fig. S9 BOMD simulations of (a) C_i $C_{50}B_{54}$ (**1**) at 1500K, (b) S_{10} $C_{50}B_{54}$ (**3**) at 1500K, and (c) C_s B_{184} (**9**) at 500 K, with the calculated average root−mean−square−deviations (RMSD) and maximum bond length deviations (MAXD) indicated.

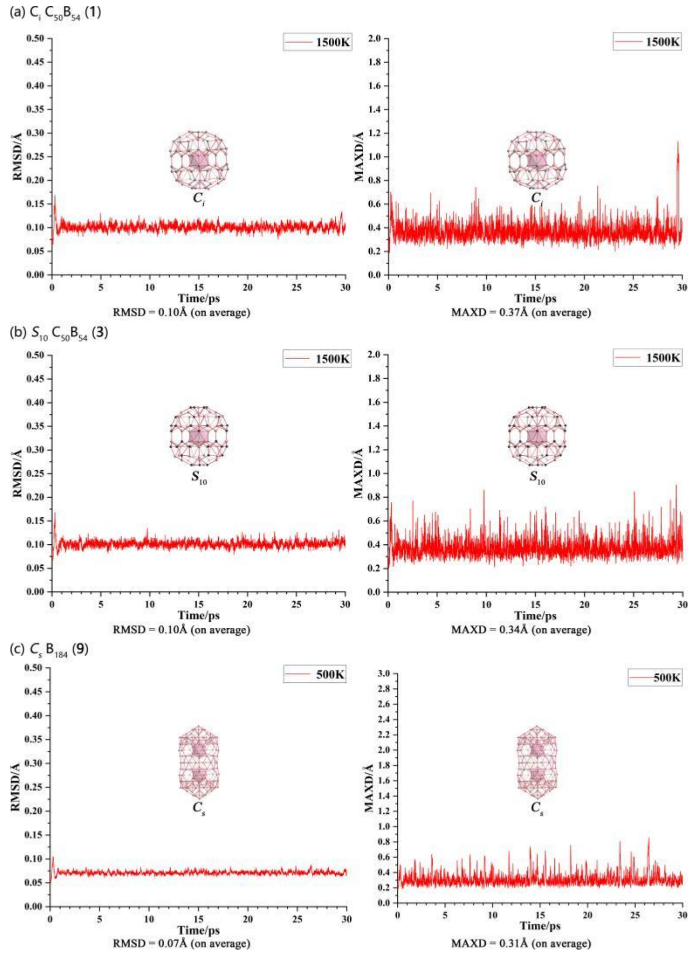

Fig. S10 The superatomic electronic configuration $1S^21P^61D^{10}1F^8$ of carborane D_{5d} $C_2B_{10}H_{12}$ in canonical molecular orbitals.

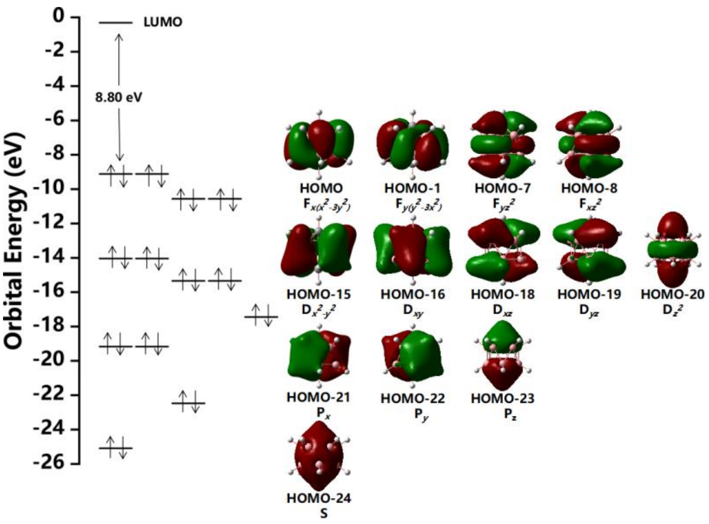

Fig. S11 AdNDP bonding pattern of S_{10} $C_{50}B_{54}$ (**3**) with the occuoation numbers indicated.

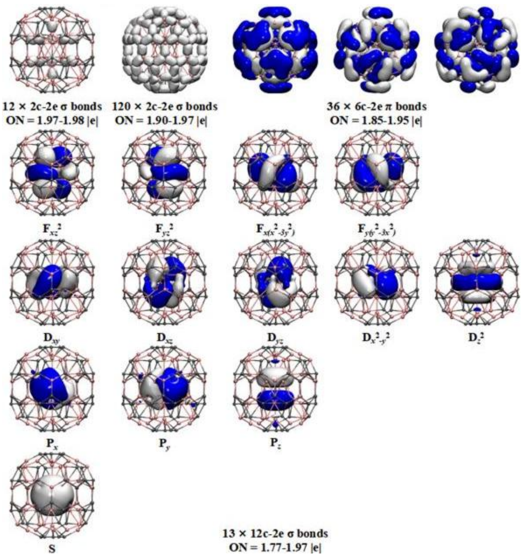

Fig. S12 The superatomic bonding orbitals of the two icosahedral B_{12} cores in C_s B_{182} (**8**) and C_s B_{184} (**9**) revealed by AdNDP analyses.

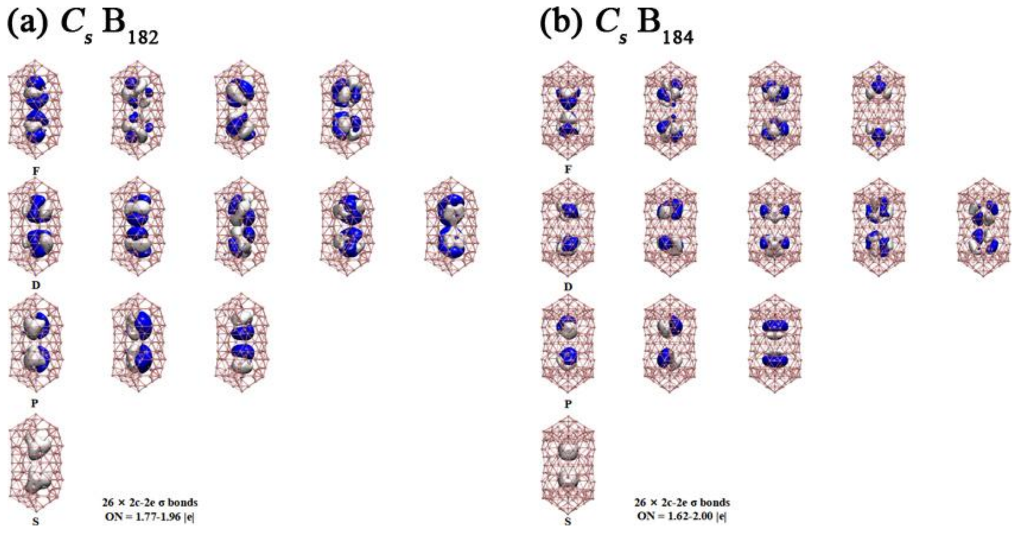

Fig. S13 Isosurfaces of the electron densities of delocalized bonds (EDDB) of C_i $C_{50}B_{54}$ (**1**) (a) and C_s $C_{78}B_{88}$ (**4**) (b), with the isovalues and average values of atomic contribution indicated.

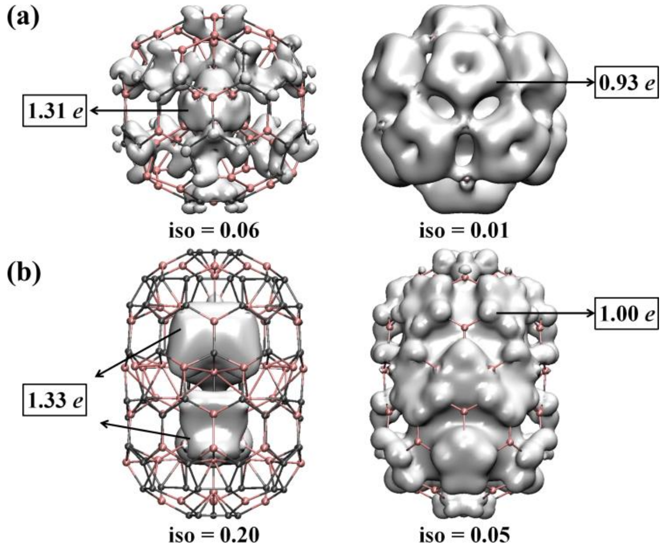

Table S1 Calculated formation energies (E_f) (in eV/atom) and HOMO-LUMO gaps (ΔE_{gap}) (in eV) of the optimized borafullerenes $C_{50}B_{54}$ (**1, 2, 3**) and $C_{88}B_{78}$ (**4, 5, 6**) and the calculated NICS values (in ppm) at the geometric centers of the icosahedral-C_nB_{12-n} cores in them at PBE0/6-311G(d).

**Cluster(Symmetry)**	**PBE0**		
	$\boldsymbol{E_f}$	**NICS**	$\boldsymbol{\Delta E_{gap}}$
$C_{50}B_{54}(C_i)$ (**1**)	-0.213	-23.23	2.28
$C_{50}B_{54}(C_1)$ (**2**)	-0.211	-20.54	2.24
$C_{50}B_{54}(S_{10})$ (**3**)	-0.207	-17.70	2.75
$C_{88}B_{78}(C_s)$ (**4**)	-0.209	-32.47/-28.04	0.83
$C_{88}B_{78}(C_s)$ (**5**)	-0.204	-29.06/-28.25	1.75
$C_{88}B_{78}(C_s)$ (**6**)	-0.189	-28.39/-28.66	1.61

Table S2 Calculated cohesive energies per atom (E_c) (in eV/atom) and HOMO-LUMO gaps (ΔE_{gap}) (in eV) of the optimized core-shell borospherenes B_n (n = 176-188) and the calculated NICS values (in ppm) at the geometric centers of icosahedral-B_{12} cores in them at PBE0/6-311G(d) level.

Cluster(Symmetry)	PBE0		
	$-E_c$	NICS	ΔE_{gap}
$B_{176}(C_1)$	5.668	-29.52/-29.52	1.51
$B_{178}(C_1)$	5.678	-27.80/-27.80	1.35
$B_{180}(C_s)$ (**7**)	5.681	-30.95/-30.95	1.46
$B_{182}(C_s)$ (**8**)	5.679	-28.32/-28.32	1.32
$B_{184}(C_s)$ (**9**)	5.691	-32.68/-32.65	0.87
$B_{186}(C_{5v})$	5.670	-20.63/-20.63	1.21
$B_{188}(C_s)$	5.673	-25.27/-25.27	1.02

Table S3 Optimized coordinates (x, y, z) of C_i $C_{50}B_{54}$ (**1**), C_1 $C_{50}B_{54}$ (**2**), S_{10} $C_{50}B_{54}$ (**3**), C_s $C_{88}B_{78}$ (**4**), C_s $C_{88}B_{78}$ (**5**), C_s $C_{88}B_{78}$ (**6**), C_s B_{180} (**7**), C_s B_{182} (**8**) and C_s B_{184} (**9**) at PBE0/6-311G(d) level.

C_i $C_{50}B_{54}$ (**1**)

B	-0.04757300	2.86537500	-1.50473900
B	0.04757300	-2.86537500	1.50473900
B	1.70314700	-2.30417700	-1.50872400
B	0.00741500	-0.04178400	-3.10049800
B	-2.73228100	0.83475500	-1.50881300
B	-1.70314700	2.30417700	1.50872400
B	1.62589300	2.35749700	1.50822200
B	2.70281200	0.92886000	-1.50668200
B	2.73228100	-0.83475500	1.50881300
B	-0.00741500	0.04178400	3.10049800
B	-2.70281200	-0.92886000	1.50668200
B	-1.62589300	-2.35749700	-1.50822200
B	1.37316300	0.47381000	-0.73625500
B	0.82905000	1.19949500	0.73446400
B	-1.38979600	0.41840700	-0.73908100
B	-0.87746700	1.16594200	0.73361600
B	0.87746700	-1.16594200	-0.73361600
B	1.38979600	-0.41840700	0.73908100
B	-0.82905000	-1.19949500	-0.73446400
B	-1.37316300	-0.47381000	0.73625500
B	-0.02855300	1.45361700	-0.74123500
B	0.02855300	-1.45361700	0.74123500
B	1.64979800	-2.19260100	3.53030700

B	2.58364300	0.88590900	3.55307300
B	-0.06259700	2.87195700	3.61467800
B	-1.55676000	-2.26107500	3.53182200
B	-2.61505900	0.77509100	3.55800800
C	0.02360800	4.37113600	-0.77048100
B	2.54513900	3.42856400	-0.80818200
B	-2.47227800	3.48189600	-0.80855500
C	4.15261300	1.32772800	-0.76970800
B	4.03602800	-1.36077000	-0.81508100
C	2.54602700	-3.54722200	-0.76836200
B	-0.04576100	-4.28932300	-0.81206800
C	-2.58262200	-3.51601300	-0.76924800
B	-4.06428200	-1.27487500	-0.81468900
C	-4.13727500	1.37197500	-0.76951600
B	-4.03602800	1.36077000	0.81508100
C	-2.54602700	3.54722200	0.76836200
B	0.04576100	4.28932300	0.81206800
C	2.58262200	3.51601300	0.76924800
B	4.06428200	1.27487500	0.81468900
C	4.13727500	-1.37197500	0.76951600
B	2.47227800	-3.48189600	0.80855500
C	-0.02360800	-4.37113600	0.77048100
B	-2.54513900	-3.42856400	0.80818200
C	-4.15261300	-1.32772800	0.76970800
B	0.06259700	-2.87195700	-3.61467800
B	2.61505900	-0.77509100	-3.55800800
B	-2.58364300	-0.88590900	-3.55307300
B	1.55676000	2.26107500	-3.53182200
B	-1.64979800	2.19260100	-3.53030700
C	1.23704900	-0.31060800	-4.21874400
C	-1.20392000	-0.42042400	-4.19539900
C	0.69171600	1.06696100	-4.16815600
C	-0.78197300	1.00081500	-4.16135700
C	1.40075300	-3.26885700	-2.85595200
B	2.70160800	-2.39088200	-2.96579200
B	-1.43194600	-3.37053800	-2.96201100
C	-2.61085000	-2.32567900	-2.85960400
C	3.49869600	0.33979300	-2.85576100
C	1.36198600	-3.95539100	-1.55037300
C	-1.41800500	-4.01445800	-1.54547100
C	3.35020600	-2.57304300	-1.55640500
C	-3.30478400	-2.49796200	-1.56173200
C	4.15540600	0.07687800	-1.55670700
B	3.10986900	1.85152100	-2.95855300

B	-3.61250700	0.33644800	-2.96309700
C	-3.01964600	1.78483200	-2.85252100
C	0.76056400	3.44260300	-2.85138700
B	-0.79596900	3.54745800	-2.95754000
C	3.48845600	2.40368000	-1.55135400
C	-4.23592500	0.11116200	-1.55215200
C	-3.40213000	2.38366500	-1.55352900
C	-1.20613600	4.07022300	-1.54988300
C	1.21413500	3.98507500	-1.55284800
C	-1.21413500	-3.98507500	1.55284800
C	-0.76056400	-3.44260300	2.85138700
C	-3.48845600	-2.40368000	1.55135400
B	-3.10986900	-1.85152100	2.95855300
C	1.20613600	-4.07022300	1.54988300
B	0.79596900	-3.54745800	2.95754000
C	3.40213000	-2.38366500	1.55352900
C	3.01964600	-1.78483200	2.85252100
C	4.23592500	-0.11116200	1.55215200
B	3.61250700	-0.33644800	2.96309700
C	-4.15540600	-0.07687800	1.55670700
C	-3.49869600	-0.33979300	2.85576100
C	2.61085000	2.32567900	2.85960400
C	3.30478400	2.49796200	1.56173200
C	-3.35020600	2.57304300	1.55640500
B	-2.70160800	2.39088200	2.96579200
C	-1.36198600	3.95539100	1.55037300
C	-1.40075300	3.26885700	2.85595200
B	1.43194600	3.37053800	2.96201100
C	1.41800500	4.01445800	1.54547100
C	-1.23704900	0.31060800	4.21874400
C	1.20392000	0.42042400	4.19539900
C	0.78197300	-1.00081500	4.16135700
C	-0.69171600	-1.06696100	4.16815600
C	-0.00285900	0.00662900	1.55030000
C	0.00285900	-0.00662900	-1.55030000
B	-0.06195200	1.32720300	4.34461400
B	0.06195200	-1.32720300	-4.34461400

C$_1$ $C_{50}B_{54}$ (**2**)

B	-0.84588100	2.82247900	-1.27265700
B	0.84483900	-2.83127300	1.32939700
B	2.33700000	-1.55950000	-1.54872500
B	0.06878900	0.24936900	-3.13193600

B	-2.82038300	0.12534400	-1.53359100
B	-2.35810100	1.54599500	1.61022500
B	0.85197300	2.58195400	1.76058600
B	2.33938200	1.78167900	-1.28187800
B	2.82744400	-0.13114400	1.58100600
B	-0.10961400	-0.25360300	3.10881900
B	-2.36780500	-1.76967800	1.34731400
B	-0.85114200	-2.58508400	-1.70081700
B	0.03285600	0.12393800	-1.56035800
B	1.18498100	0.91081200	-0.59598000
B	0.42039800	1.31737500	0.88794400
B	-1.44167400	0.05083100	-0.72774800
B	-1.20576200	0.78770300	0.80618400
B	1.19316800	-0.79422500	-0.73199800
B	1.43464400	-0.05877200	0.80000300
B	-0.43117300	-1.32550900	-0.81082600
B	-1.20035800	-0.91379200	0.67182000
B	-0.44402700	1.43250400	-0.59234200
B	0.43114000	-1.43923000	0.66811500
B	2.15646400	-1.88123300	3.45210900
B	2.15085000	1.31739600	3.70464200
B	-0.91415600	2.31389300	3.74119000
B	-0.90492100	-2.87682600	3.33290200
B	-2.95730400	-0.28978400	3.54795400
C	-1.22833300	4.24420700	-0.47076300
B	1.45837500	4.05778600	-0.45408200
B	-3.34839400	2.67102200	-0.67054700
C	3.62303000	2.53078300	-0.50704900
B	4.27720800	-0.07258400	-0.72771800
C	3.49271200	-2.59666600	-0.91816000
B	1.20970900	-4.01483700	-1.10148900
C	-1.43973700	-4.04831100	-1.13567900
B	-3.50371700	-2.32005900	-1.07333400
C	-4.36061800	0.18146900	-0.87158300
B	-4.30525700	0.07415500	0.72082300
C	-3.47281700	2.58477000	0.90272500
B	-1.21108000	4.01631700	1.11205100
C	1.43594800	4.02756400	1.13039400
B	3.51514900	2.32633000	1.07104400
C	4.34195200	-0.17771200	0.85083500
B	3.36283000	-2.67528100	0.66971000
C	1.22679700	-4.22354400	0.46897100
B	-1.45839500	-4.06137600	0.46276200
C	-3.60582100	-2.51753400	0.49160300

B	0.92432500	-2.29770200	-3.73155000
B	2.79709200	0.28522800	-3.48615400
B	-2.12052900	-1.31590900	-3.72062300
B	0.91054400	2.86501200	-3.32092500
B	-2.12930000	1.87704500	-3.46707800
C	0.51489100	-0.83676500	-4.34383800
C	1.34396200	0.37473000	-4.22966200
C	-0.89779000	-0.42256800	-4.34146800
C	0.44348400	1.53749800	-4.15617600
C	-0.94194500	1.04474200	-4.22575800
C	2.31475600	-2.42117100	-3.00033900
B	3.33677500	-1.25018600	-2.99244100
B	-0.33456700	-3.32797900	-3.23539400
C	-1.76266600	-2.72659000	-3.11370000
C	3.27817500	1.54082300	-2.66481100
C	2.46459500	-3.23013800	-1.76622600
C	-0.15747800	-4.09552900	-1.88916600
C	3.99965000	-1.36860000	-1.58710900
C	-2.40990700	-3.21294900	-1.87054400
C	3.98029000	1.38654600	-1.36772800
B	2.48624300	2.87806300	-2.68112300
B	-3.45707000	-0.48043400	-3.08180100
C	-3.31999800	1.05157500	-2.85043900
C	-0.20169600	3.68739200	-2.56817600
B	-1.71155900	3.35574800	-2.73486800
C	2.67718500	3.42012800	-1.23234100
C	-4.05081000	-0.99037700	-1.73367100
C	-3.90731200	1.41772700	-1.53736800
C	-2.29657800	3.65524500	-1.32137900
C	0.04373400	4.25713200	-1.21931000
C	-0.03837500	-4.26665700	1.22777100
C	0.21084200	-3.73009200	2.58620300
C	-2.67098700	-3.40782400	1.23325700
B	-2.51507400	-2.88392500	2.70144700
C	2.30267500	-3.64900300	1.31925600
B	1.73033600	-3.38015400	2.74880000
C	3.92385800	-1.41770500	1.53069800
C	3.37237200	-1.05937500	2.85783300
C	4.05805600	0.99307600	1.72163800
B	3.50589800	0.48338100	3.08980700
C	-4.01413600	-1.39490600	1.35695900
C	-3.36803200	-1.55776900	2.67781200
C	1.78689200	2.74874300	3.13097700
C	2.41714500	3.21387300	1.87088700

C	-4.01856300	1.37573400	1.57227800
B	-3.41481000	1.26755800	3.00750200
C	-2.46412000	3.22308800	1.77302100
C	-2.34195500	2.43048300	3.02357900
B	0.34554400	3.36333500	3.26026400
C	0.16131800	4.09591300	1.89270200
C	-0.48832800	0.87673300	4.28455300
C	0.92705000	0.42777500	4.24624000
C	0.96413800	-1.04493600	4.13622700
C	-0.42700100	-1.56423500	4.11429500
C	-0.04317000	-0.12748200	1.57467400
B	-1.42470300	-0.37934700	4.31002900

S_{10} $C_{50}B_{54}$ (**3**)

B	-0.05475100	2.84779700	-1.49818200
B	0.05475100	-2.84779700	1.49818200
B	1.71862100	-2.27274500	-1.49580200
B	0.00044200	-0.00123200	-3.16113300
B	-2.72565800	0.82773800	-1.49771100
B	-1.71862100	2.27274500	1.49580200
B	1.62963700	2.33703200	1.49643200
B	2.69192100	0.93143700	-1.49740300
B	2.72565800	-0.82773800	1.49771100
B	-0.00044200	0.00123200	3.16113300
B	-2.69192100	-0.93143700	1.49740300
B	-1.62963700	-2.33703200	-1.49643200
B	0.00025900	-0.00089300	-1.59316000
B	1.37228500	0.48637400	-0.72679500
B	0.82416000	1.20087600	0.72664900
B	-1.39623500	0.41261600	-0.72739800
B	-0.88725600	1.15519000	0.72627000
B	0.88725600	-1.15519000	-0.72627000
B	1.39623500	-0.41261600	0.72739800
B	-0.00025900	0.00089300	1.59316000
B	-0.82416000	-1.20087600	-0.72664900
B	-1.37228500	-0.48637400	0.72679500
B	-0.03871100	1.45540000	-0.72754200
B	0.03871100	-1.45540000	0.72754200
B	1.65180300	-2.16964300	3.56776200
B	2.57370400	0.89992900	3.56805300
B	-0.06073900	2.72548300	3.56818300
B	-1.55323100	-2.24111500	3.56776500
B	-2.61109900	0.78422500	3.56795800

C	0.02702500	4.36970000	-0.78530700
B	2.54548600	3.42061000	-0.80673900
B	-2.46643700	3.47799700	-0.80652600
C	4.16447900	1.32457600	-0.78536300
B	4.03971700	-1.36408900	-0.80687400
C	2.54712500	-3.55207700	-0.78567900
B	-0.04912900	-4.26352700	-0.80703100
C	-2.59078100	-3.52003000	-0.78561100
B	-4.06993100	-1.27087000	-0.80697500
C	-4.14777600	1.37602400	-0.78537900
B	-4.03971700	1.36408900	0.80687400
C	-2.54712500	3.55207700	0.78567900
B	0.04912900	4.26352700	0.80703100
C	2.59078100	3.52003000	0.78561100
B	4.06993100	1.27087000	0.80697500
C	4.14777600	-1.37602400	0.78537900
B	2.46643700	-3.47799700	0.80652600
C	-0.02702500	-4.36970000	0.78530700
B	-2.54548600	-3.42061000	0.80673900
C	-4.16447900	-1.32457600	0.78536300
B	0.06073900	-2.72548300	-3.56818300
B	2.61109900	-0.78422500	-3.56795800
B	-2.57370400	-0.89992900	-3.56805300
B	1.55323100	2.24111500	-3.56776500
B	-1.65180300	2.16964300	-3.56776200
C	0.06190100	-1.25241200	-4.28956400
C	1.21040900	-0.32794000	-4.28925600
C	-1.17216600	-0.44575400	-4.28885300
C	0.68608200	1.04993900	-4.28847400
C	-0.78629500	0.97713800	-4.28836400
C	1.39012000	-3.20082100	-2.86060100
B	2.70796400	-2.37753300	-2.96546400
B	-1.42458000	-3.31037700	-2.96577900
C	-2.61477500	-2.31131800	-2.86056700
C	3.47438200	0.33319800	-2.86091700
C	1.34854600	-3.92214300	-1.56083500
C	-1.41369900	-3.99518800	-1.56045500
C	3.36268500	-2.57909900	-1.56022800
C	-3.31315600	-2.49456600	-1.56060300
C	4.14698100	0.07058700	-1.56078800
B	3.09863000	1.84102900	-2.96548300
B	-3.58923600	0.33197900	-2.96596700
C	-3.00690500	1.77267300	-2.86069700
C	0.75678900	3.40751900	-2.86106000

B	-0.79358900	3.51606900	-2.96585200
C	3.49154100	2.40083800	-1.55983800
C	-4.23626500	0.10981100	-1.56031300
C	-3.39631000	2.38005300	-1.56033200
C	-1.20459900	4.06248300	-1.55993200
C	1.21410100	3.96554400	-1.56067200
C	-1.21410100	-3.96554400	1.56067200
C	-0.75678900	-3.40751900	2.86106000
C	-3.49154100	-2.40083800	1.55983800
B	-3.09863000	-1.84102900	2.96548300
C	1.20459900	-4.06248300	1.55993200
B	0.79358900	-3.51606900	2.96585200
C	3.39631000	-2.38005300	1.56033200
C	3.00690500	-1.77267300	2.86069700
C	4.23626500	-0.10981100	1.56031300
B	3.58923600	-0.33197900	2.96596700
C	-4.14698100	-0.07058700	1.56078800
C	-3.47438200	-0.33319800	2.86091700
C	2.61477500	2.31131800	2.86056700
C	3.31315600	2.49456600	1.56060300
C	-3.36268500	2.57909900	1.56022800
B	-2.70796400	2.37753300	2.96546400
C	-1.34854600	3.92214300	1.56083500
C	-1.39012000	3.20082100	2.86060100
B	1.42458000	3.31037700	2.96577900
C	1.41369900	3.99518800	1.56045500
C	-1.21040900	0.32794000	4.28925600
C	-0.06190100	1.25241200	4.28956400
C	1.17216600	0.44575400	4.28885300
C	0.78629500	-0.97713800	4.28836400
C	-0.68608200	-1.04993900	4.28847400

C_s $C_{88}B_{78}$ (**4**)

B	2.37681800	-0.69364500	1.63956300
B	-2.25899600	-3.70315500	-1.59616400
B	-0.90205100	-0.71698000	-2.84336400
B	-0.90205100	-0.71698000	2.84336400
B	0.86666900	-3.72741000	2.71229500
B	2.92498600	-3.66312400	0.00000000
B	2.37681800	-0.69364500	-1.63956300
B	0.86666900	-3.72741000	-2.71229500
B	-0.00599500	-5.27294100	0.00000000
B	-2.25899600	-3.70315500	1.59616400
B	-2.82674900	-0.66302100	0.00000000

B	1.22462000	-1.50564400	-0.86156000
B	1.49195700	-2.95717900	0.00000000
B	-0.42271600	-1.44682000	1.42104000
B	0.47965500	-2.94113000	1.38983100
B	-0.42271600	-1.44682000	-1.42104000
B	0.47965500	-2.94113000	-1.38983100
B	-1.43491400	-1.48245500	0.00000000
B	-1.12827600	-2.91594600	0.84818300
B	1.22462000	-1.50564400	0.86156000
B	-1.12827600	-2.91594600	-0.84818300
B	1.75769100	0.91282900	-2.55307800
B	-0.87679400	3.68744100	2.64668200
B	1.75769100	0.91282900	2.55307800
B	-2.64111600	0.77906500	-1.17105100
B	-0.87679400	3.68744100	-2.64668200
B	2.21750500	3.77748600	-1.63617400
B	2.92169000	0.59713400	0.00000000
B	2.21750500	3.77748600	1.63617400
B	-0.02609300	5.25765600	0.00000000
B	-2.78520300	3.68846700	0.00000000
B	-2.64111600	0.77906500	1.17105100
B	1.51095500	1.45392100	0.00000000
B	1.17090800	2.92856600	-0.84211400
B	-1.12319800	1.40940200	-0.88809400
B	-0.41874700	2.87012900	-1.38737600
B	0.57884900	1.44434500	1.45116400
B	1.17090800	2.92856600	0.84211400
B	-1.12319800	1.40940200	0.88809400
B	-1.41058300	2.88076400	0.00000000
B	0.57884900	1.44434500	-1.45116400
B	-0.41874700	2.87012900	1.38737600
C	-1.25222300	-6.43072300	0.00000000
C	-4.37885200	3.03586800	0.00000000
B	1.27930500	3.05604000	4.02666400
B	4.11874800	3.08346800	0.00000000
B	1.27930500	3.05604000	-4.02666400
B	-3.40068000	3.01454000	-2.43441700
B	-3.40068000	3.01454000	2.43441700
B	-4.41728200	0.81131400	-1.34447000
B	-4.41728200	0.81131400	1.34447000
C	-4.25784300	-1.53916200	0.00000000
B	-3.46286400	-1.53737800	2.51113800
C	-3.87677000	-3.86689600	1.34629300
C	-3.87677000	-3.86689600	-1.34629300

C	-2.43166000	-3.79948600	-3.30103300
B	-3.46286400	-1.53737800	-2.51113800
B	-2.46570700	0.73803600	-3.47935300
B	-0.06593200	0.74852600	-4.34356600
B	2.77767800	0.85565500	-3.85267700
B	1.30611600	-1.50423800	-4.04587600
C	2.37381100	-3.82825300	-3.42160200
C	4.03740600	-3.86268700	-1.22983400
C	-2.43166000	-3.79948600	3.30103300
B	-2.46570700	0.73803600	3.47935300
B	-0.06593200	0.74852600	4.34356600
B	2.77767800	0.85565500	3.85267700
B	4.16594800	0.84886800	1.45419800
B	4.16594800	0.84886800	-1.45419800
B	4.46198700	-1.42703700	0.00000000
B	1.30611600	-1.50423800	4.04587600
C	2.37381100	-3.82825300	3.42160200
C	4.03740600	-3.86268700	1.22983400
C	1.21912800	6.40566100	0.00000000
B	-2.17873500	5.70124000	1.56385700
B	0.77088700	5.73921800	2.53394500
B	-2.17873500	5.70124000	-1.56385700
B	2.62072100	5.75661900	0.00000000
B	0.77088700	5.73921800	-2.53394500
B	-0.77882200	-5.73044300	-2.52476100
B	2.15755800	-5.68375100	-1.55889700
B	2.15755800	-5.68375100	1.55889700
B	-2.64423200	-5.76703300	0.00000000
B	-0.77882200	-5.73044300	2.52476100
B	0.00502200	-3.85169500	-4.24359500
B	0.00502200	-3.85169500	4.24359500
C	-0.00218600	3.71302500	0.00000000
C	0.07071600	0.69741300	0.00000000
C	0.04377800	-0.74459800	0.00000000
C	0.03012800	-3.73988600	0.00000000
C	-2.19084600	-5.07826900	-2.56219200
C	-2.19084600	-5.07826900	2.56219200
C	0.33510800	-5.13778500	-3.42134900
C	0.33510800	-5.13778500	3.42134900
C	1.76044600	-5.09676200	-2.96313500
C	1.76044600	-5.09676200	2.96313500
C	-3.09390300	-5.12067300	-1.35304600
C	-3.09390300	-5.12067300	1.35304600
C	3.37058700	-5.08848100	-0.74476200

C	3.37058700	-5.08848100	0.74476200
C	-0.37323400	-6.42288400	-1.18921700
C	1.02083300	-6.40207500	-0.73031300
C	1.02083300	-6.40207500	0.73031300
C	-0.37323400	-6.42288400	1.18921700
C	0.34460500	6.39635900	-1.20214800
C	-1.06343700	6.37820800	-0.74187300
C	-1.06343700	6.37820800	0.74187300
C	0.34460500	6.39635900	1.20214800
C	-3.34011100	5.06710400	-0.74185500
C	-3.34011100	5.06710400	0.74185500
C	-1.77114400	5.05536100	-2.93087900
C	-1.77114400	5.05536100	2.93087900
C	-0.35752300	5.07038200	-3.40067400
C	-0.35752300	5.07038200	3.40067400
C	2.20838800	5.13558900	-2.56826800
C	2.20838800	5.13558900	2.56826800
C	3.09587200	5.13609100	1.36281500
C	3.09587200	5.13609100	-1.36281500
C	-3.96008600	3.79177400	-1.18809600
C	-3.96008600	3.79177400	1.18809600
C	-2.37564500	3.76667400	-3.36928400
C	-2.37564500	3.76667400	3.36928400
C	-0.11630900	3.79232400	-4.12995800
C	-0.11630900	3.79232400	4.12995800
C	3.84805800	3.84505600	-1.36928800
C	3.84805800	3.84505600	1.36928800
C	2.44326900	3.85766100	-3.29889800
C	2.44326900	3.85766100	3.29889800
C	-1.36174500	2.99954200	-4.11667100
C	-1.36174500	2.99954200	4.11667100
C	3.44487000	3.08865700	-2.57228000
C	3.44487000	3.08865700	2.57228000
C	-3.47751100	1.45424900	-2.42446400
C	-3.47751100	1.45424900	2.42446400
C	-1.37557900	1.53607400	-4.19063300
C	-1.37557900	1.53607400	4.19063300
C	1.36243300	1.49152500	-4.15211400
C	1.36243300	1.49152500	4.15211400
C	-4.60757500	1.60161500	0.00000000
C	3.49827200	1.61977100	-2.67610600
C	3.49827200	1.61977100	2.67610600
C	4.23247700	1.53491500	0.00000000
C	-4.12311200	-0.75235000	-1.25925600

C	-4.12311200	-0.75235000	1.25925600
C	-2.42621000	-0.87365900	-3.46667100
C	-2.42621000	-0.87365900	3.46667100
C	-0.11807600	-0.86818000	-4.24201400
C	-0.11807600	-0.86818000	4.24201400
C	2.53026000	-0.66941000	-3.39089200
C	2.53026000	-0.66941000	3.39089200
C	4.11145700	-0.70372900	-1.39086800
C	4.11145700	-0.70372900	1.39086800
C	-1.40112200	-1.68203200	-4.18258000
C	-1.40112200	-1.68203200	4.18258000
C	3.48343300	-1.50386200	-2.49532800
C	3.48343300	-1.50386200	2.49532800
B	-4.16758900	-3.11744700	0.00000000
B	4.60194100	-3.09079700	0.00000000
C	-3.47887700	-3.07957000	-2.54852200
C	-3.47887700	-3.07957000	2.54852200
C	-1.37969900	-3.12571700	-4.07838900
C	-1.37969900	-3.12571700	4.07838900
C	1.36561700	-3.05842000	4.16611100
C	1.36561700	-3.05842000	-4.16611100
B	3.42179100	-3.07690300	-2.47191800
B	3.42179100	-3.07690300	2.47191800

C_s $C_{88}B_{78}$ (**5**)

B	-2.53453500	0.73142100	1.81262100
B	2.22397100	3.60041700	-1.62355200
B	0.88534900	0.67110600	-2.85870900
B	0.88534900	0.67110600	2.85870900
B	-0.80939600	3.70106700	2.68816900
B	-2.74965800	3.72097800	0.00000000
B	-2.53453500	0.73142100	-1.81262100
B	-0.80939600	3.70106700	-2.68816900
B	0.05058300	5.33951600	0.00000000
B	2.22397100	3.60041700	1.62355200
B	2.82190300	0.51618300	0.00000000
B	-1.27545100	1.35220700	-0.88777600
B	-1.47520500	2.82151900	0.00000000
B	0.40498500	1.35461800	1.42106100
B	-0.49393600	2.83173800	1.39494700
B	0.40498500	1.35461800	-1.42106100
B	-0.49393600	2.83173800	-1.39494700
B	-0.03496800	3.72463800	0.00000000

B	1.44681700	1.39179800	0.00000000
B	1.11411800	2.81371400	0.85068600
B	-1.27545100	1.35220700	0.88777600
B	1.11411800	2.81371400	-0.85068600
B	-0.93810700	-1.22104100	-2.87524200
B	0.82983800	-3.72499200	2.65077400
B	-0.93810700	-1.22104100	2.87524200
B	2.64232300	-0.88367700	-1.19372500
B	0.82983800	-3.72499200	-2.65077400
B	-2.29383700	-3.67422900	-1.63769000
B	-3.02453400	-1.18103800	0.00000000
B	-2.29383700	-3.67422900	1.63769000
B	-0.06843800	-5.41257500	0.00000000
B	2.76384100	-3.78274600	0.00000000
B	2.64232300	-0.88367700	1.19372500
B	-1.46517700	-1.52526200	0.00000000
B	-1.15991300	-2.96107600	-0.84049400
B	1.17987300	-1.52299200	-0.86243400
B	0.43906400	-2.96023900	-1.35801900
B	-0.44769800	-1.52462400	1.39609900
B	-1.15991300	-2.96107600	0.84049400
B	-0.01027100	-3.85365300	0.00000000
B	1.17987300	-1.52299200	0.86243400
B	1.42450900	-2.97566200	0.00000000
B	-0.44769800	-1.52462400	-1.39609900
B	0.43906400	-2.96023900	1.35801900
C	-0.88095300	6.54857700	-0.73333600
C	-0.88095300	6.54857700	0.73333600
C	0.51674600	6.48149300	1.18178200
C	-1.64380400	5.16109200	2.87919400
C	-3.17991000	5.18627900	0.74337200
C	-3.17991000	5.18627900	-0.74337200
C	-1.64380400	5.16109200	-2.87919400
C	-0.22320900	5.13493600	-3.35729200
C	2.30279800	4.98792100	-2.53732800
C	3.16347300	4.95841100	-1.30626100
C	3.16347300	4.95841100	1.30626100
C	2.30279800	4.98792100	2.53732800
C	-0.22320900	5.13493600	3.35729200
B	-1.34862700	-3.02284000	4.03816800
B	-4.28410700	-2.97205000	0.00000000
B	-1.34862700	-3.02284000	-4.03816800
B	3.36028300	-3.11733100	-2.43592500
B	3.36028300	-3.11733100	2.43592500

B	4.43006300	-0.91256600	-1.32618900
B	4.43006300	-0.91256600	1.32618900
B	3.46019600	1.42608400	2.48600700
C	3.50654300	2.94720100	2.49163000
C	3.87625400	3.67095300	1.25142500
C	3.87625400	3.67095300	-1.25142500
C	3.50654300	2.94720100	-2.49163000
C	2.50256200	3.72465900	-3.27696500
B	3.46019600	1.42608400	-2.48600700
B	2.49047800	-0.80286000	-3.49665100
B	0.11290400	-0.70690600	-4.40793100
B	-2.68975900	-0.65666300	-3.53152600
B	-1.33127200	1.60636900	-4.07824400
C	1.42179400	3.09225900	-4.06415300
C	-1.36198400	3.14234600	-4.14232000
C	-3.43146200	3.33864600	-2.50518500
C	-2.35745300	3.94386400	-3.32049500
C	-3.85054800	3.97699200	-1.24046500
C	-3.43146200	3.33864600	2.50518500
C	-1.36198400	3.14234600	4.14232000
C	1.42179400	3.09225900	4.06415300
C	2.50256200	3.72465900	3.27696500
B	2.49047800	-0.80286000	3.49665100
B	0.11290400	-0.70690600	4.40793100
B	-2.68975900	-0.65666300	3.53152600
B	-4.20577600	-0.62797800	1.45136100
B	-4.20577600	-0.62797800	-1.45136100
B	-4.31367700	1.65403300	0.00000000
B	-1.33127200	1.60636900	4.07824400
C	-2.35745300	3.94386400	3.32049500
C	-3.85054800	3.97699200	1.24046500
B	2.04286400	-5.80019600	1.55750100
B	-0.91448200	-5.72302700	2.51823200
B	2.04286400	-5.80019600	-1.55750100
B	-2.74079600	-5.68185400	0.00000000
B	-0.91448200	-5.72302700	-2.51823200
B	0.90281600	5.70456100	-2.49125200
B	-2.01393100	5.81349600	-1.53758300
B	-2.01393100	5.81349600	1.53758300
B	2.70962100	5.63756500	0.00000000
B	0.90281600	5.70456100	2.49125200
B	0.03417400	3.87144000	-4.21636000
B	0.03417400	3.87144000	4.21636000
C	-0.01366000	-0.76680600	0.00000000

C	-0.05045500	0.65852900	0.00000000
C	0.91540400	-6.53017700	-0.75010300
C	0.91540400	-6.53017700	0.75010300
C	-0.50842300	-6.48784500	1.20965200
C	-0.50842300	-6.48784500	-1.20965200
C	0.51674600	6.48149300	-1.18178200
C	-4.30656100	3.21989700	0.00000000
C	-1.38268100	-6.46370800	0.00000000
C	3.24031100	-5.18589600	-0.74217700
C	3.24031100	-5.18589600	0.74217700
C	1.65975800	-5.12694300	-2.93309400
C	1.65975800	-5.12694300	2.93309400
C	0.23695800	-5.08809200	-3.39061800
C	0.23695800	-5.08809200	3.39061800
C	-2.33160400	-5.03909300	-2.56403400
C	-2.33160400	-5.03909300	2.56403400
C	-3.20898900	-5.02016300	-1.35063400
C	-3.20898900	-5.02016300	1.35063400
C	3.90914700	-3.92157000	-1.19252800
C	3.90914700	-3.92157000	1.19252800
C	2.30682700	-3.85020500	-3.37786600
C	2.30682700	-3.85020500	3.37786600
C	0.03000100	-3.80293500	-4.12289800
C	0.03000100	-3.80293500	4.12289800
C	-2.54224600	-3.74631000	-3.28774700
C	-2.54224600	-3.74631000	3.28774700
C	-3.94503300	-3.72268000	-1.34938600
C	-3.94503300	-3.72268000	1.34938600
C	1.30276900	-3.05446000	-4.12279200
C	1.30276900	-3.05446000	4.12279200
C	-3.54383400	-2.95153400	-2.54310100
C	-3.54383400	-2.95153400	2.54310100
C	-4.45949700	-1.40447300	0.00000000
C	3.48338400	-1.54507200	-2.43110100
C	3.48338400	-1.54507200	2.43110100
C	1.35955000	-1.58507300	-4.20613800
C	1.35955000	-1.58507300	4.20613800
C	-1.38872600	-1.44932000	-4.22961600
C	-1.38872600	-1.44932000	4.22961600
C	-3.63030800	-1.48331600	-2.61602500
C	-3.63030800	-1.48331600	2.61602500
C	4.36694600	-3.17233000	0.00000000
C	4.62922500	-1.73816800	0.00000000
C	4.14737700	0.67141100	-1.24250400

C	4.14737700	0.67141100	1.24250400
C	2.42371300	0.79183500	-3.46817100
C	2.42371300	0.79183500	3.46817100
C	0.11403300	0.89531500	-4.28822300
C	0.11403300	0.89531500	4.28822300
C	-2.60821400	0.91184300	-3.45735300
C	-2.60821400	0.91184300	3.45735300
C	-4.12824200	0.95643400	-1.41322100
C	-4.12824200	0.95643400	1.41322100
C	4.17033600	2.97286000	0.00000000
C	1.36817300	6.44527100	0.00000000
C	4.28504200	1.48330100	0.00000000
C	1.41585400	1.65647300	-4.18357300
C	1.41585400	1.65647300	4.18357300
B	-3.67029400	1.80777600	-2.66860400
B	-3.67029400	1.80777600	2.66860400

C_s $C_{88}B_{78}$ (**6**)

B	2.44124900	-0.74059300	1.76571200
B	-2.21271100	-3.66686300	-1.61758500
B	-0.90575800	-0.74731600	-2.85422100
B	-0.90575800	-0.74731600	2.85422100
B	0.79418100	-3.72310800	2.68074100
B	2.72132500	-3.72554800	0.00000000
B	2.44124900	-0.74059300	-1.76571200
B	0.79418100	-3.72310800	-2.68074100
B	-0.02485200	-5.35174500	0.00000000
B	-2.21271100	-3.66686300	1.61758500
B	-2.86373600	-0.58211300	0.00000000
B	-0.00752600	-0.69065700	0.00000000
B	1.21537700	-1.38326100	-0.87822000
B	1.45216300	-2.84026000	0.00000000
B	-0.44770800	-1.40438900	1.42361700
B	0.47028400	-2.86349700	1.39732800
B	-0.44770800	-1.40438900	-1.42361700
B	0.47028400	-2.86349700	-1.39732800
B	0.02735700	-3.74980000	0.00000000
B	-1.49053900	-1.44494900	0.00000000
B	-1.13432900	-2.86307000	0.84807100
B	1.21537700	-1.38326100	0.87822000
B	-1.13432900	-2.86307000	-0.84807100
B	0.78302800	1.31442600	-2.89974200
B	-0.82579700	3.78539700	2.64825000

B	0.78302800	1.31442600	2.89974200
B	-2.79901000	1.02026300	-0.96765600
B	-0.82579700	3.78539700	-2.64825000
B	2.28732300	3.68807000	-1.63759300
B	2.95731700	1.25670800	0.00000000
B	2.28732300	3.68807000	1.63759300
B	0.09162500	5.44107900	0.00000000
B	-2.72643900	3.88406700	0.00000000
B	-2.79901000	1.02026300	0.96765600
B	-0.08942800	0.81067000	0.00000000
B	1.40302900	1.52948400	0.00000000
B	1.15429100	2.97482700	-0.84623700
B	-1.26136300	1.62397200	-0.85247300
B	-0.45054200	3.02671300	-1.36059300
B	0.37664700	1.56204400	1.40192200
B	1.15429100	2.97482700	0.84623700
B	0.03915900	3.89313300	0.00000000
B	-1.26136300	1.62397200	0.85247300
B	-1.40704300	3.08435200	0.00000000
B	0.37664700	1.56204400	-1.40192200
B	-0.45054200	3.02671300	1.36059300
C	-0.46679600	-6.50083700	-1.18286600
C	0.93203300	-6.54314100	-0.73231400
C	-1.32236400	-6.47813600	0.00000000
C	0.93203300	-6.54314100	0.73231400
C	-0.46679600	-6.50083700	1.18286600
C	3.94411400	3.72504000	1.34989700
C	2.54269300	3.75182400	-3.29083300
C	-2.30568600	3.85977200	-3.38652000
C	-3.89249300	3.94215100	1.18985700
C	-0.02806700	3.81585900	4.13017600
C	-1.29725700	3.06225200	-4.11806200
C	-4.27885100	3.18261700	0.00000000
C	-1.29725700	3.06225200	4.11806200
C	3.54357800	2.95920000	2.54699500
C	3.54357800	2.95920000	-2.54699500
C	1.68829200	-5.13737600	2.88678700
C	3.22970200	-5.14113600	0.74796700
C	3.22970200	-5.14113600	-0.74796700
C	1.68829200	-5.13737600	-2.88678700
C	0.26354900	-5.14956600	-3.37337900
C	-2.27890600	-5.03577000	-2.54738400
C	-3.14538700	-5.01762200	-1.31092200
C	-3.14538700	-5.01762200	1.31092200

C	-2.27890600	-5.03577000	2.54738400
C	0.26354900	-5.14956600	3.37337900
C	1.39171600	1.43204700	-4.22056300
C	-3.51935200	1.53335100	-2.47343900
C	-3.51935200	1.53335100	2.47343900
C	1.39171600	1.43204700	4.22056300
C	4.41254700	1.37845700	0.00000000
C	-1.66764100	5.15690000	2.95577800
C	-0.23845500	5.11785800	3.41334700
C	-3.26276300	5.23312400	0.74948400
C	-3.26276300	5.23312400	-0.74948400
C	2.33609200	5.04966800	2.56977200
C	3.21285400	5.02802200	1.35340500
C	-1.66764100	5.15690000	-2.95577800
C	-0.23845500	5.11785800	-3.41334700
C	3.21285400	5.02802200	-1.35340500
C	2.33609200	5.04966800	-2.56977200
B	1.34980900	3.02522000	4.04248700
B	4.27295600	2.96791300	0.00000000
B	1.34980900	3.02522000	-4.04248700
B	-3.36525700	3.11555700	-2.44573900
B	-3.36525700	3.11555700	2.44573900
B	-4.43500100	0.85563600	-1.39898300
B	-4.43500100	0.85563600	1.39898300
C	-4.17620400	-0.72053200	-1.25562900
C	-4.17620400	-0.72053200	1.25562900
C	-4.32384900	-1.52247700	0.00000000
B	-3.46038700	-1.46275400	2.48343600
C	-4.16036200	-3.02193500	0.00000000
C	-3.49045900	-2.99400900	2.49173200
C	-3.86437000	-3.72415000	1.25343000
C	-3.86437000	-3.72415000	-1.25343000
C	-3.49045900	-2.99400900	-2.49173200
C	-2.49005500	-3.76133300	-3.28043400
B	-3.46038700	-1.46275400	-2.48343600
C	-2.43172100	-0.81045000	-3.47565100
B	-2.50175700	0.79745000	-3.49810800
B	-0.11371800	0.70378500	-4.43321200
B	2.66456100	0.66717200	-3.50542800
C	2.59645300	-0.93181600	-3.39354500
C	3.56378800	-1.77277600	-2.59180800
B	1.31495100	-1.60581100	-4.06233000
C	-0.11770800	-0.89967900	-4.29026600
C	-1.42329100	-1.67261000	-4.20603500

C	-1.41783100	-3.11322500	-4.07410500
C	1.35970100	-3.13168500	-4.13864300
C	3.39035500	-3.21824400	-2.47406300
C	4.29338200	-3.16853700	0.00000000
C	2.35420500	-3.88582400	-3.31952600
C	3.85259900	-3.89878000	-1.22908100
C	3.39035500	-3.21824400	2.47406300
C	1.35970100	-3.13168500	4.13864300
C	-1.41783100	-3.11322500	4.07410500
C	-2.49005500	-3.76133300	3.28043400
B	-2.50175700	0.79745000	3.49810800
C	-2.43172100	-0.81045000	3.47565100
C	-1.42329100	-1.67261000	4.20603500
B	-0.11371800	0.70378500	4.43321200
C	-0.11770800	-0.89967900	4.29026600
B	2.66456100	0.66717200	3.50542800
C	2.59645300	-0.93181600	3.39354500
B	4.15653600	0.63695800	1.45671400
C	4.05097500	-0.96558700	1.42279000
B	4.15653600	0.63695800	-1.45671400
C	4.05097500	-0.96558700	-1.42279000
B	4.26678600	-1.63633300	0.00000000
C	3.56378800	-1.77277600	2.59180800
B	1.31495100	-1.60581100	4.06233000
C	2.35420500	-3.88582400	3.31952600
C	3.85259900	-3.89878000	1.22908100
C	-0.90961700	6.55274800	0.75116100
C	0.51403500	6.51460800	1.21167700
C	-0.90961700	6.55274800	-0.75116100
C	1.39130100	6.49420900	0.00000000
C	0.51403500	6.51460800	-1.21167700
B	-2.04623600	5.82934500	1.56907000
B	0.91658400	5.73957000	2.52605000
B	-2.04623600	5.82934500	-1.56907000
B	2.74441700	5.69257300	0.00000000
B	0.91658400	5.73957000	-2.52605000
C	3.94411400	3.72504000	-1.34989700
C	2.54269300	3.75182400	3.29083300
C	-2.30568600	3.85977200	3.38652000
C	-0.02806700	3.81585900	-4.13017600
C	-3.89249300	3.94215100	-1.18985700
C	3.62413200	1.48870200	2.61933800
C	3.62413200	1.48870200	-2.61933800
C	-1.34935900	1.58263200	-4.19811000

C	-4.36592900	1.68538700	0.00000000
C	-1.34935900	1.58263200	4.19811000
B	-0.86563000	-5.72989400	-2.49855500
B	2.05645100	-5.78339200	-1.53819300
B	2.05645100	-5.78339200	1.53819300
B	-2.68234000	-5.68754100	0.00000000
B	-0.86563000	-5.72989400	2.49855500
B	-0.02664500	-3.88325900	-4.22268800
B	-0.02664500	-3.88325900	4.22268800

C_s B_{180} (**7**)

B	-0.89190900	1.10069300	3.52493500
B	0.82802000	1.12036800	3.56590000
B	1.37029700	-0.50752000	3.58774400
B	-1.41410400	-0.54281100	3.55579700
B	-0.00503400	-1.52676000	3.58510300
B	1.45401000	0.41340100	2.05248400
B	0.94482100	-1.29105800	2.05448500
B	-1.44575200	0.35418500	2.01980100
B	-0.82229500	-1.31837900	2.05827900
B	-0.03240500	-0.05907400	4.50429700
B	0.03308300	-0.08319500	0.95212400
B	-1.73987100	3.83176100	4.04018100
B	-3.19993100	2.77899300	3.97368600
B	-3.20138700	1.93906100	5.49949000
B	-1.75801400	2.35909200	6.39260700
B	-0.90297100	3.48451800	5.50055600
B	0.75027200	1.13070700	7.30807200
B	-1.50961400	-0.50458000	7.30343200
B	-3.74809200	0.31997300	5.50690200
B	-2.88207700	-0.91384100	6.40026400
B	-0.12481800	-1.51245700	7.24900300
B	-1.52191700	-3.40438700	5.49462900
B	-4.32704300	-0.37684100	4.08960600
B	-1.91365100	-3.71653100	2.75627100
B	-3.49588600	-2.88371700	2.66820800
B	-1.40503500	-2.82861300	0.00000000
B	-3.87333400	-2.11223500	1.28706400
B	-2.83122500	-1.81038900	0.00000000
B	-4.40197600	-0.38353000	1.18592600
B	-4.53200300	0.52871800	2.57043400
B	-3.93328500	2.14160300	2.57194600
B	-3.11966200	2.74013600	1.21815400

B	-3.19837800	-0.08722000	0.00000000
B	-2.96503200	1.64075900	0.00000000
B	-0.74075200	-3.43157000	1.42879600
B	-0.98072300	-4.23461900	4.05121900
B	0.86935900	-3.92529000	1.31687900
B	1.85577600	-4.07112500	2.69529200
B	1.81089900	-3.59032500	0.00000000
B	-0.63778200	3.82328500	0.00000000
B	-1.69606000	3.71624700	1.25318600
B	-0.83524300	4.31647600	2.65063300
B	0.95226300	3.84848800	0.00000000
B	3.21217000	-2.80285200	0.00000000
B	3.68829800	-2.10997400	1.49476100
B	4.15995500	-0.49514800	1.48148200
B	4.09800400	0.37810300	0.00000000
B	3.62728200	1.93920400	0.00000000
B	3.15489900	2.68905300	1.46808900
B	1.77876200	3.65485700	1.47629800
B	3.94417000	2.09780500	2.80164600
B	4.44831800	0.46744800	2.80973200
B	4.17322600	-0.53090900	4.21466000
B	3.61680100	-2.18023600	4.20238100
B	3.27655900	-3.10469100	2.78466700
B	1.27741100	-3.46718300	5.56085300
B	2.67537400	-2.42398300	5.62284200
B	2.60745500	-0.94922600	6.48316000
B	1.27465500	-0.50826900	7.34662900
B	-0.14855800	-2.92330500	6.37929300
B	3.56894600	0.19710000	5.65291000
B	1.59009500	2.25379300	6.45141400
B	3.03382200	1.84745500	5.63676700
B	3.12417500	2.76857600	4.18387300
B	0.82102100	3.49269900	5.56904700
B	0.88861000	4.29771400	2.71340600
B	1.72170000	3.79172300	4.14187600
B	0.79054600	-4.20777700	4.08609800
B	-0.10408400	-0.03048300	6.21420000
B	-3.91841100	-2.08634300	4.12300500
B	-2.85612700	-2.37118500	5.47754000
B	-0.11357700	-2.80024400	4.60524700
B	-2.11985200	-2.54951800	1.48692400
B	-2.68484800	-0.85806000	4.58992500
B	-2.95602600	0.88073800	1.51775800
B	-1.67053000	2.13354900	4.57402500

B	-0.00763000	3.04977100	1.43923300
B	1.54985200	2.12727400	4.68056500
B	3.00062500	0.89660500	1.50862800
B	2.51478300	-0.89503600	4.72397100
B	1.94062200	-2.55143100	1.48764700
B	-0.09987700	-0.07199100	8.14506400
B	4.08449200	-1.44519300	5.67185100
B	2.52932000	3.48470200	5.62520400
B	-2.64731300	3.53394400	5.42590300
B	-4.27917500	-1.32753800	5.56051900
B	-0.16421200	-4.42918700	5.51741800
B	-0.00687200	1.44288600	2.02239000
B	-0.89190900	1.10069300	-3.52493500
B	-1.41410400	-0.54281100	-3.55579700
B	-0.00503400	-1.52676000	-3.58510300
B	0.82802000	1.12036800	-3.56590000
B	1.37029700	-0.50752000	-3.58774400
B	-0.82229500	-1.31837900	-2.05827900
B	0.94482100	-1.29105800	-2.05448500
B	-0.00687200	1.44288600	-2.02239000
B	1.45401000	0.41340100	-2.05248400
B	-0.03240500	-0.05907400	-4.50429700
B	0.03308300	-0.08319500	-0.95212400
B	-3.19993100	2.77899300	-3.97368600
B	-1.73987100	3.83176100	-4.04018100
B	-0.90297100	3.48451800	-5.50055600
B	-1.75801400	2.35909200	-6.39260700
B	-3.20138700	1.93906100	-5.49949000
B	-0.94284600	1.17906000	-7.32652800
B	-1.50961400	-0.50458000	-7.30343200
B	0.75027200	1.13070700	-7.30807200
B	0.82102100	3.49269900	-5.56904700
B	1.59009500	2.25379300	-6.45141400
B	1.27465500	-0.50826900	-7.34662900
B	3.56894600	0.19710000	-5.65291000
B	1.72170000	3.79172300	-4.14187600
B	4.44831800	0.46744800	-2.80973200
B	3.94417000	2.09780500	-2.80164600
B	3.15489900	2.68905300	-1.46808900
B	1.77876200	3.65485700	-1.47629800
B	0.88861000	4.29771400	-2.71340600
B	-0.83524300	4.31647600	-2.65063300
B	-1.69606000	3.71624700	-1.25318600
B	4.15995500	-0.49514800	-1.48148200

B	4.17322600	-0.53090900	-4.21466000
B	3.68829800	-2.10997400	-1.49476100
B	3.27655900	-3.10469100	-2.78466700
B	-3.11966200	2.74013600	-1.21815400
B	-3.93328500	2.14160300	-2.57194600
B	0.86935900	-3.92529000	-1.31687900
B	-0.74075200	-3.43157000	-1.42879600
B	-3.87333400	-2.11223500	-1.28706400
B	-4.40197600	-0.38353000	-1.18592600
B	-3.49588600	-2.88371700	-2.66820800
B	-1.91365100	-3.71653100	-2.75627100
B	-0.98072300	-4.23461900	-4.05121900
B	0.79054600	-4.20777700	-4.08609800
B	1.85577600	-4.07112500	-2.69529200
B	2.67537400	-2.42398300	-5.62284200
B	1.27741100	-3.46718300	-5.56085300
B	-0.14855800	-2.92330500	-6.37929300
B	-0.12481800	-1.51245700	-7.24900300
B	2.60745500	-0.94922600	-6.48316000
B	-1.52191700	-3.40438700	-5.49462900
B	-2.88207700	-0.91384100	-6.40026400
B	-2.85612700	-2.37118500	-5.47754000
B	-3.91841100	-2.08634300	-4.12300500
B	-3.74809200	0.31997300	-5.50690200
B	-4.53200300	0.52871800	-2.57043400
B	-4.32704300	-0.37684100	-4.08960600
B	3.61680100	-2.18023600	-4.20238100
B	-0.10408400	-0.03048300	-6.21420000
B	3.12417500	2.76857600	-4.18387300
B	3.03382200	1.84745500	-5.63676700
B	2.51478300	-0.89503600	-4.72397100
B	3.00062500	0.89660500	-1.50862800
B	1.54985200	2.12727400	-4.68056500
B	-0.00763000	3.04977100	-1.43923300
B	-1.67053000	2.13354900	-4.57402500
B	-2.95602600	0.88073800	-1.51775800
B	-2.68484800	-0.85806000	-4.58992500
B	-2.11985200	-2.54951800	-1.48692400
B	-0.11357700	-2.80024400	-4.60524700
B	1.94062200	-2.55143100	-1.48764700
B	-0.09987700	-0.07199100	-8.14506400
B	-0.16421200	-4.42918700	-5.51741800
B	-4.27917500	-1.32753800	-5.56051900
B	-2.64731300	3.53394400	-5.42590300

B	2.52932000	3.48470200	-5.62520400
B	4.08449200	-1.44519300	-5.67185100
B	-1.44575200	0.35418500	-2.01980100
B	-0.94284600	1.17906000	7.32652800
B	-2.24830400	0.70837600	6.32787400
B	-0.01142100	4.17411200	4.05952100
B	2.39264200	-3.49827600	4.12687500
B	3.96382200	1.21106300	4.18911600
B	-4.31596700	-1.26625200	2.60998000
B	4.09127400	-1.39168400	0.00000000
B	2.54325100	3.33069600	0.00000000
B	-4.31596700	-1.26625200	-2.60998000
B	2.39264200	-3.49827600	-4.12687500
B	3.96382200	1.21106300	-4.18911600
B	-0.01142100	4.17411200	-4.05952100
B	-2.24830400	0.70837600	-6.32787400

C_s B_{182} (**8**)

B	-1.42342200	-0.08552400	3.61659300
B	-0.43677900	1.32829300	3.62442100
B	1.21473900	0.85757800	3.55961700
B	-0.40491400	-1.46885400	3.52296800
B	1.25020200	-0.88272200	3.44741900
B	0.41681200	1.45144600	2.04525700
B	1.48033500	0.05825100	1.97119200
B	-1.22998400	-0.91337100	2.02994900
B	0.44698600	-1.41678400	1.96067700
B	0.07393300	-0.08159800	4.52186700
B	-0.04197800	0.01913700	0.94595200
B	-4.18219100	0.79036100	4.29896600
B	-4.16667500	-1.00624800	4.29245900
B	-3.27640100	-1.51645200	5.64673300
B	-2.81125900	-0.12775700	6.52265800
B	-3.31683600	1.27778200	5.68455400
B	-0.39995200	1.30432300	7.36115200
B	-0.39199000	-1.48142300	7.34958900
B	-2.30154400	-2.87135900	5.54951900
B	-0.81564300	-2.84441900	6.40729700
B	1.28349700	-0.92032200	7.36447900
B	2.01910700	-3.09036200	5.50500200
B	-2.11928900	-3.84489400	4.14574600
B	2.28948400	-3.80910600	2.70556200
B	0.56853600	-4.26337600	2.76750000

B	1.78554800	-2.66052000	0.00000000
B	-0.47244200	-4.03653500	1.41006600
B	0.33918200	-3.69472500	0.00000000
B	-2.17836000	-3.63584600	1.45199400
B	-3.03955000	-3.30483000	2.80940400
B	-4.06080900	-1.90307100	2.80824600
B	-4.19082200	-0.97281500	1.44262400
B	-2.86059100	-3.04981800	0.00000000
B	-3.80797700	-1.72964400	0.00000000
B	2.89263700	-3.05277100	1.43504600
B	3.01057400	-3.19760700	4.13010300
B	3.89634400	-1.54487700	1.45371300
B	4.48508400	-0.74560400	2.72262400
B	3.17206500	-0.68642000	0.00000000
B	-3.76489300	1.62286400	0.00000000
B	-4.16172600	0.85977200	1.45701400
B	-4.06685200	1.75357000	2.83915000
B	-2.83024700	2.94917100	0.00000000
B	3.52960900	1.01942800	0.00000000
B	3.79913300	1.83459600	1.37575200
B	2.69217200	3.25859400	1.37527600
B	1.83837500	3.58967300	0.00000000
B	0.29835400	4.06789100	0.00000000
B	-0.52866000	4.22560000	1.45288100
B	-2.25869700	3.58789300	1.45934800
B	0.39424200	4.41334000	2.80583600
B	2.08571100	3.91843100	2.77322100
B	2.80571200	3.26170200	4.23319700
B	3.93745600	1.86773700	4.18096600
B	4.33965200	1.03698900	2.70905600
B	3.59021400	-0.76769400	5.52248800
B	3.58047600	0.89899200	5.55800300
B	2.35958800	1.67057000	6.45810300
B	1.24892700	0.79839500	7.30493800
B	2.44869200	-1.68187200	6.42063600
B	1.85761800	3.08689200	5.63676700
B	-0.88384400	2.63979800	6.52905900
B	0.26515700	3.56815500	5.66001200
B	-0.49385100	4.21307800	4.29307800
B	-2.37394300	2.64435900	5.68110900
B	-3.06763900	3.19011100	2.83338500
B	-2.20232400	3.61741400	4.29756900
B	4.08190500	-1.66886100	4.13543900
B	0.08837000	-0.11000100	6.23500900

B	-0.38445600	-4.39459500	4.12683400
B	0.39227400	-3.70726600	5.52027000
B	2.32215600	-1.56513500	4.58696700
B	0.95788300	-3.01338900	1.49992600
B	-0.77082800	-2.73722300	4.51903400
B	-2.52381700	-1.90737900	1.63244200
B	-2.68168200	-0.10565400	4.72226600
B	-2.55080200	1.76079400	1.67854100
B	-0.85175900	2.51426800	4.73038800
B	0.92865800	2.96770600	1.56874600
B	2.23157100	1.62125100	4.64508300
B	3.11380800	0.19808100	1.51484100
B	0.07023000	-0.03913200	8.16556000
B	3.49491800	2.60022200	5.65863000
B	-1.37680000	4.02537000	5.75057300
B	-4.28289800	-0.13027400	5.74577800
B	-1.28684700	-4.24658300	5.52255400
B	3.66521900	-2.50426300	5.52794200
B	-1.23206600	0.84366300	2.07815800
B	-1.42342200	-0.08552400	-3.61659300
B	-0.40491400	-1.46885400	-3.52296800
B	1.25020200	-0.88272200	-3.44741900
B	-0.43677900	1.32829300	-3.62442100
B	1.21473900	0.85757800	-3.55961700
B	0.44698600	-1.41678400	-1.96067700
B	1.48033500	0.05825100	-1.97119200
B	-1.23206600	0.84366300	-2.07815800
B	0.41681200	1.45144600	-2.04525700
B	0.07393300	-0.08159800	-4.52186700
B	-0.04197800	0.01913700	-0.94595200
B	-4.16667500	-1.00624800	-4.29245900
B	-4.18219100	0.79036100	-4.29896600
B	-3.31683600	1.27778200	-5.68455400
B	-2.81125900	-0.12775700	-6.52265800
B	-3.27640100	-1.51645200	-5.64673300
B	-1.38546100	-0.11215900	-7.33236700
B	-0.39199000	-1.48142300	-7.34958900
B	-0.39995200	1.30432300	-7.36115200
B	-2.37394300	2.64435900	-5.68110900
B	-0.88384400	2.63979800	-6.52905900
B	1.24892700	0.79839500	-7.30493800
B	1.85761800	3.08689200	-5.63676700
B	-2.20232400	3.61741400	-4.29756900
B	2.08571100	3.91843100	-2.77322100

B	0.39424200	4.41334000	-2.80583600
B	-0.52866000	4.22560000	-1.45288100
B	-2.25869700	3.58789300	-1.45934800
B	-3.06763900	3.19011100	-2.83338500
B	-4.06685200	1.75357000	-2.83915000
B	-4.16172600	0.85977200	-1.45701400
B	2.69217200	3.25859400	-1.37527600
B	2.80571200	3.26170200	-4.23319700
B	3.79913300	1.83459600	-1.37575200
B	4.33965200	1.03698900	-2.70905600
B	-4.19082200	-0.97281500	-1.44262400
B	-4.06080900	-1.90307100	-2.80824600
B	3.89634400	-1.54487700	-1.45371300
B	2.89263700	-3.05277100	-1.43504600
B	-0.47244200	-4.03653500	-1.41006600
B	-2.17836000	-3.63584600	-1.45199400
B	0.56853600	-4.26337600	-2.76750000
B	2.28948400	-3.80910600	-2.70556200
B	3.01057400	-3.19760700	-4.13010300
B	4.08190500	-1.66886100	-4.13543900
B	4.48508400	-0.74560400	-2.72262400
B	3.58047600	0.89899200	-5.55800300
B	3.59021400	-0.76769400	-5.52248800
B	2.44869200	-1.68187200	-6.42063600
B	1.28349700	-0.92032200	-7.36447900
B	2.35958800	1.67057000	-6.45810300
B	2.01910700	-3.09036200	-5.50500200
B	-0.81564300	-2.84441900	-6.40729700
B	0.39227400	-3.70726600	-5.52027000
B	-0.38445600	-4.39459500	-4.12683400
B	-2.30154400	-2.87135900	-5.54951900
B	-3.03955000	-3.30483000	-2.80940400
B	-2.11928900	-3.84489400	-4.14574600
B	3.93745600	1.86773700	-4.18096600
B	0.08837000	-0.11000100	-6.23500900
B	-0.49385100	4.21307800	-4.29307800
B	0.26515700	3.56815500	-5.66001200
B	2.23157100	1.62125100	-4.64508300
B	0.92865800	2.96770600	-1.56874600
B	-0.85175900	2.51426800	-4.73038800
B	-2.55080200	1.76079400	-1.67854100
B	-2.68168200	-0.10565400	-4.72226600
B	-2.52381700	-1.90737900	-1.63244200
B	-0.77082800	-2.73722300	-4.51903400

B	0.95788300	-3.01338900	-1.49992600
B	2.32215600	-1.56513500	-4.58696700
B	3.11380800	0.19808100	-1.51484100
B	0.07023000	-0.03913200	-8.16556000
B	3.66521900	-2.50426300	-5.52794200
B	-1.28684700	-4.24658300	-5.52255400
B	-4.28289800	-0.13027400	-5.74577800
B	-1.37680000	4.02537000	-5.75057300
B	3.49491800	2.60022200	-5.65863000
B	-1.22998400	-0.91337100	-2.02994900
B	-1.38546100	-0.11215900	7.33236700
B	0.79877000	-2.33848900	6.52065300
B	-1.36578700	3.99341900	2.88594100
B	-4.26464000	-0.12310200	2.88350100
B	2.81209400	-1.95083200	2.88602900
B	3.40424100	2.69513300	2.76025800
B	-1.35018400	3.81127000	0.00000000
B	-4.04680500	-0.07268800	0.00000000
B	-1.39873000	-3.89907800	0.00000000
B	3.41849100	-2.32867300	0.00000000
B	2.81209400	-1.95083200	-2.88602900
B	3.40424100	2.69513300	-2.76025800
B	-1.36578700	3.99341900	-2.88594100
B	-4.26464000	-0.12310200	-2.88350100
B	0.79877000	-2.33848900	-6.52065300

C_s B_{184} (**9**)

B	-1.07928000	0.98496400	3.58414200
B	0.60438400	1.29395000	3.59779600
B	1.40955400	-0.21459800	3.60996900
B	-1.30728400	-0.72162000	3.57589000
B	0.23728600	-1.45882900	3.58361700
B	1.26532200	0.69690900	2.05045700
B	1.04193600	-1.06250100	2.03969300
B	-1.56688200	0.18506600	2.07149800
B	-0.71644600	-1.39349400	2.03179500
B	-0.02918100	-0.02451100	4.55513600
B	-0.14106700	-0.00838400	0.94834900
B	-2.43213800	3.46270200	4.16422400
B	-3.62253000	2.21160400	4.16540000
B	-3.41526500	1.29672100	5.61303000
B	-2.06656800	1.91400400	6.47137600
B	-1.51649500	3.29973600	5.61700500

B	0.60648500	1.26694200	7.36401000
B	-1.29614800	-0.74062100	7.34780600
B	-3.63992000	-0.42569500	5.60736300
B	-2.50772500	-1.39326800	6.45684300
B	0.24874300	-1.47878400	7.34063400
B	-0.76033600	-3.60240100	5.55498100
B	-4.09137600	-1.23166400	4.15402100
B	-1.13816300	-4.30572400	2.68242300
B	-2.70772300	-3.58274600	2.71904800
B	-1.10135800	-3.80344400	0.00000000
B	-3.24191000	-2.66410800	1.46760600
B	-2.61363000	-3.23477300	0.00000000
B	-4.02467300	-1.15370000	1.45594300
B	-4.51896600	-0.26890900	2.76646000
B	-4.27533400	1.42632200	2.76685200
B	-3.57923900	2.15961300	1.46284100
B	-4.10208400	-0.26767300	0.00000000
B	-3.94641200	1.37638000	0.00000000
B	-0.11892400	-4.09313800	1.29306900
B	-0.12289900	-4.22918600	4.07776100
B	1.56858000	-3.75935900	1.28846400
B	2.56156200	-3.60182400	2.69110200
B	2.45397300	-3.16798300	0.00000000
B	-1.55634900	3.73665200	0.00000000
B	-2.35628100	3.37447500	1.45501000
B	-1.65755900	4.13305100	2.75815600
B	0.07743800	4.07693100	0.00000000
B	3.78740500	-2.18945000	0.00000000
B	3.99922200	-1.37558600	1.46538800
B	4.20578500	0.34879700	1.45704300
B	4.14499200	1.21019200	0.00000000
B	3.13933700	2.56036700	0.00000000
B	2.46435500	3.15623000	1.44880900
B	0.93473600	3.95883300	1.45816300
B	3.40545500	2.80207900	2.76958300
B	4.25654800	1.33729800	2.78078900
B	4.22441800	0.30081700	4.17442800
B	3.97893300	-1.40703800	4.15539800
B	3.75775700	-2.38680200	2.75407400
B	1.95594000	-3.10606500	5.56593700
B	3.14276500	-1.83896900	5.59914000
B	2.78422100	-0.40568200	6.47425100
B	1.42011900	-0.23365100	7.35947800
B	0.50067600	-2.82836000	6.43274100

B	3.50831400	0.90217700	5.62843400
B	1.19598700	2.51338500	6.47595300
B	2.68009300	2.42848900	5.62825600
B	2.55163400	3.35451100	4.17209200
B	0.18604000	3.61421900	5.61653800
B	0.02864000	4.43063200	2.75583500
B	0.98342900	4.08306800	4.16049000
B	1.58633000	-3.93727600	4.08872900
B	-0.01357700	-0.03748300	6.27577100
B	-3.25612800	-2.74996600	4.13372300
B	-2.32967200	-2.85768800	5.57644800
B	0.49169300	-2.68134100	4.65767600
B	-1.39980900	-2.84163500	1.47741500
B	-2.40200700	-1.31697100	4.67699100
B	-3.16063300	0.41865300	1.54705700
B	-1.97951400	1.84162300	4.69007000
B	-0.59744700	3.06362100	1.55864700
B	1.15499200	2.41322300	4.68822100
B	2.79257600	1.41709800	1.52491700
B	2.67033900	-0.36324400	4.67539100
B	2.32989600	-2.16331800	1.48562000
B	-0.02458500	-0.05398800	8.19641500
B	4.31353800	-0.60476700	5.63011400
B	1.85565000	3.92389200	5.63418400
B	-3.19277300	2.98720100	5.63533000
B	-3.86953200	-2.12057700	5.60978300
B	0.77076200	-4.34410600	5.54363400
B	-0.32677100	1.47033800	2.04090500
B	-1.07928000	0.98496400	-3.58414200
B	-1.30728400	-0.72162000	-3.57589000
B	0.23728600	-1.45882900	-3.58361700
B	0.60438400	1.29395000	-3.59779600
B	1.40955400	-0.21459800	-3.60996900
B	-0.71644600	-1.39349400	-2.03179500
B	1.04193600	-1.06250100	-2.03969300
B	-0.32677100	1.47033800	-2.04090500
B	1.26532200	0.69690900	-2.05045700
B	-0.02918100	-0.02451100	-4.55513600
B	-0.14106700	-0.00838400	-0.94834900
B	-3.62253000	2.21160400	-4.16540000
B	-2.43213800	3.46270200	-4.16422400
B	-1.51649500	3.29973600	-5.61700500
B	-2.06656800	1.91400400	-6.47137600
B	-3.41526500	1.29672100	-5.61303000

B	-1.07220700	0.95711100	-7.35887900
B	-1.29614800	-0.74062100	-7.34780600
B	0.60648500	1.26694200	-7.36401000
B	0.18604000	3.61421900	-5.61653800
B	1.19598700	2.51338500	-6.47595300
B	1.42011900	-0.23365100	-7.35947800
B	3.50831400	0.90217700	-5.62843400
B	0.98342900	4.08306800	-4.16049000
B	4.25654800	1.33729800	-2.78078900
B	3.40545500	2.80207900	-2.76958300
B	2.46435500	3.15623000	-1.44880900
B	0.93473600	3.95883300	-1.45816300
B	0.02864000	4.43063200	-2.75583500
B	-1.65755900	4.13305100	-2.75815600
B	-2.35628100	3.37447500	-1.45501000
B	4.20578500	0.34879700	-1.45704300
B	4.22441800	0.30081700	-4.17442800
B	3.99922200	-1.37558600	-1.46538800
B	3.75775700	-2.38680200	-2.75407400
B	-3.57923900	2.15961300	-1.46284100
B	-4.27533400	1.42632200	-2.76685200
B	1.56858000	-3.75935900	-1.28846400
B	-0.11892400	-4.09313800	-1.29306900
B	-3.24191000	-2.66410800	-1.46760600
B	-4.02467300	-1.15370000	-1.45594300
B	-2.70772300	-3.58274600	-2.71904800
B	-1.13816300	-4.30572400	-2.68242300
B	-0.12289900	-4.22918600	-4.07776100
B	1.58633000	-3.93727600	-4.08872900
B	2.56156200	-3.60182400	-2.69110200
B	3.14276500	-1.83896900	-5.59914000
B	1.95594000	-3.10606500	-5.56593700
B	0.50067600	-2.82836000	-6.43274100
B	0.24874300	-1.47878400	-7.34063400
B	2.78422100	-0.40568200	-6.47425100
B	-0.76033600	-3.60240100	-5.55498100
B	-2.50772500	-1.39326800	-6.45684300
B	-2.32967200	-2.85768800	-5.57644800
B	-3.25612800	-2.74996600	-4.13372300
B	-3.63992000	-0.42569500	-5.60736300
B	-4.51896600	-0.26890900	-2.76646000
B	-4.09137600	-1.23166400	-4.15402100
B	3.97893300	-1.40703800	-4.15539800
B	-0.01357700	-0.03748300	-6.27577100

B	2.55163400	3.35451100	-4.17209200
B	2.68009300	2.42848900	-5.62825600
B	2.67033900	-0.36324400	-4.67539100
B	2.79257600	1.41709800	-1.52491700
B	1.15499200	2.41322300	-4.68822100
B	-0.59744700	3.06362100	-1.55864700
B	-1.97951400	1.84162300	-4.69007000
B	-3.16063300	0.41865300	-1.54705700
B	-2.40200700	-1.31697100	-4.67699100
B	-1.39980900	-2.84163500	-1.47741500
B	0.49169300	-2.68134100	-4.65767600
B	2.32989600	-2.16331800	-1.48562000
B	-0.02458500	-0.05398800	-8.19641500
B	0.77076200	-4.34410600	-5.54363400
B	-3.86953200	-2.12057700	-5.60978300
B	-3.19277300	2.98720100	-5.63533000
B	1.85565000	3.92389200	-5.63418400
B	4.31353800	-0.60476700	-5.63011400
B	-1.56688200	0.18506600	-2.07149800
B	-1.07220700	0.95711100	7.35887900
B	-0.78893800	4.08371400	4.15437300
B	-4.18002900	0.52916600	4.15825900
B	-1.84736000	-3.83074300	4.08599900
B	3.00497700	-2.90016200	4.12523700
B	3.62559700	1.95522000	4.18608100
B	4.41339900	-0.61556900	0.00000000
B	1.73855900	3.74501300	0.00000000
B	-3.09869700	2.85651700	0.00000000
B	-3.76122600	-2.03233800	0.00000000
B	-4.18002900	0.52916600	-4.15825900
B	-1.84736000	-3.83074300	-4.08599900
B	3.00497700	-2.90016200	-4.12523700
B	3.62559700	1.95522000	-4.18608100
B	-0.78893800	4.08371400	-4.15437300
B	2.27835300	-0.32239000	-0.90866000
B	2.27835300	-0.32239000	0.90866000
